# The transparency of reporting 'harms' encountered with the surgically assisted acceleration of orthodontic tooth movement in the published randomized controlled trials: a meta-epidemiological study

**DOI:** 10.1186/s40510-023-00457-4

**Published:** 2023-03-21

**Authors:** Doa’a Tahseen Alfailany, Mohammad Younis Hajeer, Khaldoun Darwich

**Affiliations:** 1 Department of Orthodontics, Faculty of Dentistry, University of Damascus, Damascus, Syria; 2 Department of Oral and Maxillofacial Surgery, Faculty of Dentistry, University of Damascus, Damascus, Syria

**Keywords:** Epidemiological studies, Acceleration, Orthodontic tooth movement, Harm, Reporting, CONSORT, Randomized controlled trials, Risks and benefits, Minimally invasive procedures

## Abstract

**Background:**

Surgical-assisted accelerated orthodontics (SAAO) has become very popular recently. Therefore, this study aimed to investigate the extent to which researchers adhere to Item 19 (harms) of the Consolidated Standards of Reporting Trials (CONSORT) in the published studies in the field of SAAO. In addition, the study evaluated the possible association between harm reporting and the human development index (HDI) of the recruited research sample country, CiteScore-based quartile (CSBQ) of the publishing journal, invasiveness of the surgical intervention (ISI), and the type of orthodontic tooth movement (TOTM). Moreover, it aimed to summarize the different possible harms and complications that maybe encountered in the course of SAAO.

**Materials and methods:**

Electronic searching of six databases was conducted for SAAO-related English RCTs published between January 2000 and April 2022. For the RCTs that did not report harms, information was sought by contacting the corresponding authors. Descriptive statistics of the evaluated variables were performed. The association between 'harm reporting' and the HDI of the research team, the BDRQ of the publication journal, the ISI, and the TOTM were investigated. Binary logistic regression was used, and the odds ratios (ORs) with 95% confidence interval (CIs) of the evaluated variables were obtained. Moreover, the risk of bias of the included RCTs was assessed using the RoB2 tool.

**Results:**

Among the 91 included RCTs, 54 RCTs (59.3%) did not adhere to reporting harm associated with the SAAO. The non-adherence was significantly associated with the ISI (OR 0.16; CI 0.03–0.73; *p* < 0.018) for invasive methods compared with minimally invasive ones). There was a significant positive correlation between harm reporting and both the CSBQ of the publishing journal and the HDI of the recruited research sample country (*p* = 0.001, *p* = 0.003, respectively). On the contrary, a non-significant association was found between harm reporting and the type of OTM (*p* = 0.695). The incidence of harms associated with SAAO was approximately 17.5%.

**Limitations:**

Assessment was restricted to English RCTs related to SAAO.

**Conclusion and implications:**

The adherence to reporting harms in the field of SAAO was deficient. Efforts should be made by authors, peer reviewers, and editors to improve compliance with the CONSORT guidelines regarding harms reporting. Additionally, there is a wide spectrum of harms that could be associated with SAAO that the practitioner should pay attention to and alert the patient to the possibility of their occurrence.

**Supplementary Information:**

The online version contains supplementary material available at 10.1186/s40510-023-00457-4.

## Introduction

Several methods for accelerating orthodontic tooth movement (OTM) have been proposed to reduce treatment time and achieve patient satisfaction [1]. Acceleration methods can be divided into conservative approaches (biomechanical, biological, and physical) and surgical ones [1].

Currently, surgical interventions can be considered one of the most applied and tested acceleration methods that have shown promising results in shortening the duration of orthodontic treatment [2, 3]. Surgical acceleration methods all rely on one principle, the 'regional acceleratory phenomenon (RAP)', which was described by Frost as a complex reaction of tissues to a harmful stimulus [4]. However, surgical interventions may be associated with many undesirable side effects such as tooth resorption as was reported by Tunçer et al. [5]. They notified exposure of the central incisors to severe cervical resorption in a patient who underwent en masse retraction following premolars extraction assisted with piezoincisions [5]. On the other hand, there have been other several reports regarding injuries associated with the surgical acceleration techniques like interdental bone loss [6, 7], tooth vitality loss [6, 8], scarring of the surgical site [9, 10], gingival recession [6, 11], mechanical root injury during surgery [12, 13], face and the neck subcutaneous hematomas [6], and bacteremia [14]. From the ethical point of view, these harms should be mentioned in the final report of any clinical study (or trial) in order to alarm all practitioners about the possibility of having these hazards to their patients when undergoing surgically assisted acceleration of tooth movement [15]. In addition, the omission of these harms may lead to misinterpretation and insufficient conclusions about the interventions evaluated [16]. Moreover, that could influence medical decision making which is based on balancing benefits against risks [17].

To formulate high-quality protocols and to enhance the quality of reported RCTs, the SPIRIT (Standard Protocol Items: Recommendations for Interventional Trials) [18], as well as the CONSORT (Consolidated Standards of Reporting Trials) statement [19], were developed. Items 22 of the SPIRIT and 19 of the CONSORT are concerned with reporting the possible risks of the intended intervention in the information sheet or the actually encountered harms in the course of the accomplished trial, respectively [19]. However, many RCTs give inadequate information on associated side effects. This lack of available information affects the transparency, and the reliability of the findings, as well as the decision to adopt the applied intervention [19]. Nowadays, few meta-epidemiological studies (MES) have touched on that item within the field of medicine [16, 20, 21]. On the other hand, regarding orthodontics, only one MES investigated the quality of reporting of RCTs abstracts in the four major orthodontic journals between 2006 and 2011 [22]. It found insufficient reporting of harms in the abstracts of the chosen RCT trials [22]. Till now, there is no MES addressing the reporting of harms in the field of surgically assisted accelerated orthodontics (SAAO). Therefore, the overall goals of our current study were (1) to investigate the extent to which researchers adhere to Item 19 (harms) of the CONSORT 2010 statement in the SAAO treatments, (2) to evaluate of the possible association or correlation between the presence/absence of 'harms' reporting in the published paper with the country of recruited research sample, the scientific strength and prestige of the publishing journal, the invasiveness of the SAAO, the type of OTM, and (3) to display the different possible harms and complications that maybe encountered in the course of SAAO.

## Materials and methods

This MES was constructed under 'Guidelines for reporting meta-epidemiological methodology research' [23].

### SAAO-related harms

Harms are defined as the totality of possible adverse consequences of an intervention or therapy. They are considered as the direct opposite of benefits, against which they must be compared [15]. In this study, the harms related to the SAAO were classified into: (1) gingival soft tissue harms such as gingival recession, gingival tearing, surgical site scarring, excessive gingival bleeding, infection in the incision site, and gingival abscess, (2) alveolar bone harms such as alveolar bone loss, alveolar bone defects such as dehiscence and fenestration, and cortical bone fracture, (3) dental harms which include loss of tooth vitality, tooth sensitivity, root resorption, mechanical root injury, (4) harms related to patient-reported outcome measures (PROMs) such as pain, discomfort, swelling or edema, difficulty in eating, restriction of jaw movement, and fear of surgical intervention, and (5) other harms such as nerve injury (e.g., numbness), and hematoma.

### Human development index (HDI)

Human Development Index (HDI) was adopted in the current study to classify the country of the recruited research sample of the included RCTs. HDI is a socio-demographic variable, which was introduced by the WHO in the 1990s [24]. It helps classify the world's population into homogeneous groups based on more comprehensive indicators (being educated, living a long and healthy life, and enjoying a decent standard of living) and not on the purely economic value of each country [24]. Countries divide into four wide groups of human development based on the numerical score obtained (ranging from 0 to 1) by the United Nations Development Programme (UNDP): 'group 1': very high HDI, 'group 2': high HDI, 'group 3': medium HDI, and 'group 4': low HDI [24].

### CiteScore™-based quartile (CSBQ)

In the current study, to evaluate the impact of the publication journal of the included research paper, the CiteScore was used [25]. The quartile-based classification was adopted. Mainly, CS-based quartiles in Scopus® were adopted to rank the journals of the included RCTs. Journals that were not indexed by Scopus®, were searched on the Web of Science™. The JIF/JCI-based quartiles in the Web of Science™ database were used on this occasion. If the journal was not found in these two major databases, it was considered 'Not indexed'.

### Type of orthodontic tooth movement (OTM)

Orthodontic tooth movement that is assisted by one of the SAAO methods includes: retraction of (canine, en-mass, or incisors), decrowding of anterior teeth, impacted canine traction, and intrusion of molar or incisors.

### Surgically assisted accelerated orthodontics (SAAO)

Surgical acceleration methods were divided into: (1) Invasive methods require full mucoperiosteal flaps, suturing with the potential associated surgical side effect [26]. It included conventional corticotomy, distraction osteogenesis which is divided into periodontal ligament distraction or dentoalveolar distraction (DAD), periodontally accelerated osteogenic orthodontics (PAOO), or any surgical technique which is required raising flap. (2) Minimally invasive methods are characterized as flapless surgical techniques with negligible risk of associated side effects [26]. It included corticision, piezocision, discision, micro-osteoperforations (MOPs), laser-assisted flapless corticotomy (LAAC), fibrotomy, interseptal bone reduction, or any surgical technique which is not required raising flap. However, some studies combine in their design two surgical acceleration methods (whether invasive vs. invasive, minimally invasive vs. minimally invasive, or invasive vs. minimally invasive), which was defined in this study as a "combination of invasive and minimally invasive procedures in a parallel group or split-mouth study designs".

### Eligibility criteria

Research articles were screened for eligibility using the following criteria:

All randomized controlled trials that were published between January 2000 to April 2022, as well as included healthy human participants of both gender who underwent fixed orthodontic treatment assisted with one of the surgical acceleration interventions (invasive or minimally invasive) compared with a non-accelerated group, or an accelerated group with another protocol or surgical technique, with no restriction for age, type of malocclusion and racial group. On the other hand, the non-RCTs, retrospective trials, animal trials, case reports or case series, and non-English language trials were excluded. The included RCTs in which the majority of their outcomes were directly related to the assessment of harms associated with the provided intervention (i.e., in which more than 50% of the harms classified under "SAAO-related harms" were evaluated as a secondary outcome), were deemed ineligible for inclusion in our study.

### Search strategy

Electronic searching of the Cochrane Library, PubMed®, Scopus®, Web of Science™, and Google™ Scholar was conducted. Moreover, in addition to the search in Google™ Scholar to determine any relevant papers in the grey literature, a search in the OpenGrey was also performed. The systematic search was done by two authors (DTA and MYH). The search was restricted to articles published between January 2000 and April 2022. For additional sources and to ensure that no relevant research paper was left out, the reference list of the selected papers was also checked. More details about the search strategy used are given in Additional file 1: Table S1.

### Study selection and data extraction

The potential eligible trials were screened separately by two authors (DTA and MYH). In case of any conflict, the third author (KD) was consulted to reach a resolution. The selection was carried out according to the following methodology: firstly, records from each database were imported into Endnote™ X9, and then merged into a single data unit to remove duplicate records and to facilitate retrieval of related articles. Then after deleting duplicate records, the titles and abstracts of the remaining articles in the Endnote™ list were only checked. Thereafter, the full text was evaluated if reading the title and abstracts did not help in deciding the eligibility of the article, as well as if the paper appeared to fit the inclusion criteria. Data were extracted by the same two investigators, then they were organized into a pre-designed table. From each study, the following data were obtained: the first author's name, year of publication, country where the study was carried out, Human Development Index (HDI) of the country of the recruited research sample (1 = very high human development, 2 = high human development, 3 = medium human development, 4 = low human development), journal of publication, and the CiteScore (CS) of the journal, the Quartile to which the journal belongs (Q) according to Scopus® indexing (Level 1 = Q1, Level 2 = Q2, Level 3 = Q3, Level 4 = Q4, Level 5 = Unclassified); a journal was labeled 'Not indexed' when it is was not found in both Scopus® and Web of Science™ databases. In addition, the following pieces of information were gathered: the type of OTM (1 = incisor, canine, or en-mass retraction, 2 = decrowding, 3 = all other OTM (e.g., molar or incisor intrusion), invasiveness of the SAAO (1 = invasive, 2 = minimally invasive, 3 = combination of invasive and minimally invasive procedures in parallel group or split-mouth study designs), adherence to harm reporting (1 = Yes, 2 = No), and the method of reporting harms (1 = reported under a definite subheading, 2 = reported inexplicitly within the Results or Discussion sections, 3 = not reported at all).

### Information about the harms requested from the authors

For more information about the harms associated with SAAO, emails were sent to the corresponding authors of the included RCTs that did not report harm. After introducing ourselves and explaining the main purpose of this study, the authors were asked to help answer the following question: "Did any of your patients suffer harm from the surgical technique applied in accelerating orthodontic tooth movement?".

### Risk of bias assessment

The risk of bias in the included RCTs was assessed by two reviewers (DTA and MYH) using the RoB-2 tool [27]. In case of controversy, the third author (KD) was consulted to reach an agreement. Five domains were evaluated as 'low risk,' 'high risk' or 'some concern of bias' as follows: bias arising from the randomization process, bias due to deviations from intended interventions (which divided into effect of assignment to intervention, and effect of adhering to intervention), bias due to missing outcome data, bias in the measurement of the outcome, and bias in the selection of the reported result. Then, the overall risk of bias was judged as follows: “Low risk” if all fields were assessed as low risk of bias, “some concerns” if at least one field was judged as having some concerns without the presence of high risk of bias for any domain, and “high risk” if at least one or more domain were assessed as at high risk or in the case of having some concerns for multiple domains.

### Statistical analysis

Descriptive statistics including the percentage and frequencies for each studied variable were calculated. Mann–Whitney test (U) was used to compare the harm reporting and the year of publication. The association between the categorical variables was detected. To investigate the association between harm reporting and CiteScore-based quartile of the publishing journal and HDI of the recruited research sample country, the rank biserial correlation was applied by using Spearman's Rho test (r_s_). Then, the strength of the relationship according to r_s_ was defined as: 0.00–0.19 “very weak correlation”, 0.20–0.39 “weak correlation”, 0.40–0.59 “moderate correlation”, 0.60–0.79 “strong correlation”, and 0.80–1.0 “very strong correlation”. Chi-square and Fisher’s exact tests were applied to examine the association between harm reporting and both invasiveness of the SAAO, and the type of OTM. Then, the strength of the relationship was detected using Cramer's V (φ_c_) as follows: from 0.00 to less than 0.10 “negligible association”, from 0.10 to less than 0.20 “weak association”, from 0.20 to less than 0.40 “moderate association”, from 0.40 to less than 0.60 “relatively strong association”, from 0.60 to less than 0.80 “strong association”, and from 0.80 to 1.00 “very strong association”. The threshold of statistical significance was determined at an alpha value of 0.05. In addition, to investigate the prediction model of reporting harms and estimate the odds ratios (ORs), binary logistic regression analysis was used. All statistical tests were carried out using the Statistical Package for the Social Sciences, version 23.0 (IBM SPSS Corp., Armonk, NY, USA).

## Results

### Study selection and inclusion in the study

**Fig. 1:**
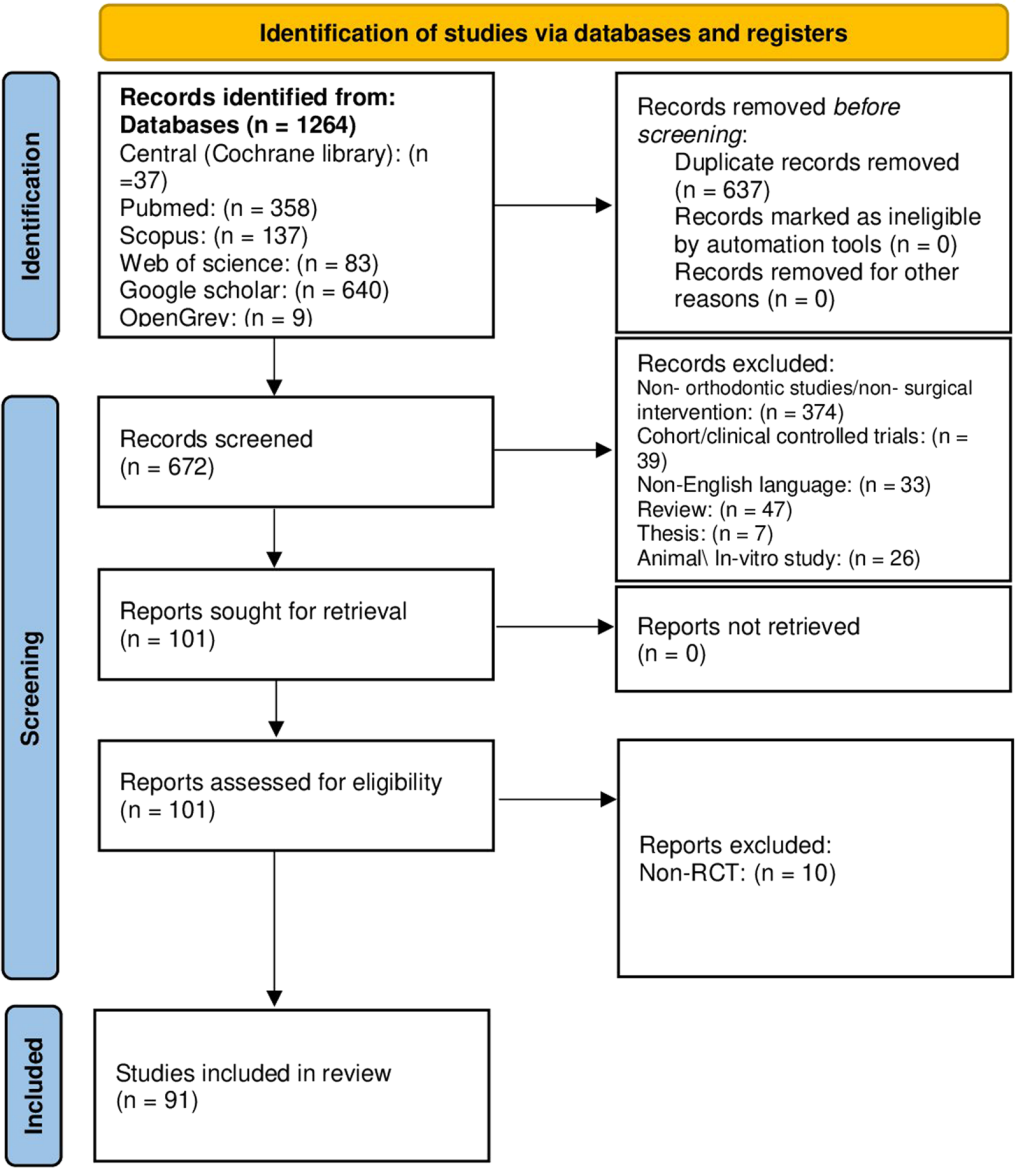
The PRISMA flow diagram of the included RCTs

In this meta-epidemiological study, a flow diagram of study selection and inclusion is given in Fig. 1. After excluding ineligible articles, 101 papers identified from six databases were reviewed in depth. No papers that met the inclusion criteria were found in the grey literature. As a result, a total of 91 RCTs in the SAAO treatments were included in this study. More details about the excluded papers and the reasons beyond exclusion are illustrated in Additional file 2: Table S2.

### Characteristics of included RCTs

**Fig. 2:**
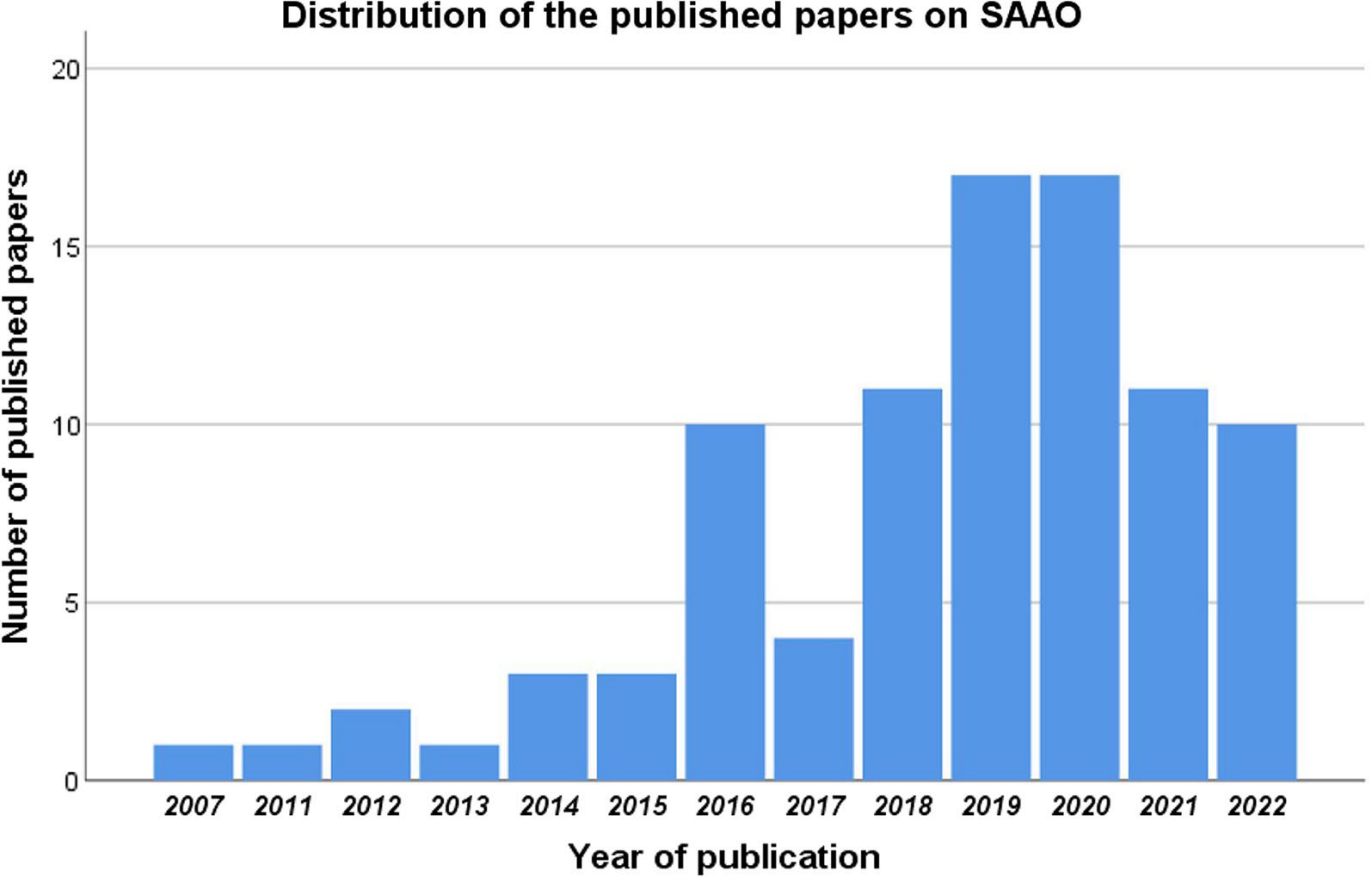
Distribution of year of publication of the included RCTs

The first SAAO-related RCT was published in 2007. Subsequently, the number of published SAAO-related studies increased dramatically from the year 2007 until 2022, where the peak of publishing was in 2019 and 2020 by 34 RCTs (37.4%) as presented in Fig. 2.

SAAO research teams of the included RCTs were distributed in seventeen countries worldwide. Moreover, nine of these countries are located in Asia. Two countries from Asia occupied the top ranks in the number of SAAO published papers, where 23 (25.3%) RCTs were conducted in India, and 15 (16.5%) SAAO trials were carried out in Syria. On the other hand, 18 (19.8%) trials were accomplished in Egypt, and 7 (7.7%) RCTs came from Turkey.

The number of SAAO papers conducted in the 'medium' human development countries was 56 trials (61.5%). In contrast, 23 RCTs (25.3%) and 12 RCTs (13.2%) were performed in both 'very high' and 'high' human development countries according to HDI data, respectively.

The American Journal of Orthodontics and Dentofacial Orthopedics, the Angle Orthodontist journal, and the European Journal of Orthodontics were the top three journals to publish SAAO-related papers, with 9, 8, and 5 published RCTs, respectively. Moreover, there were about 33 journals that published one article related to the topic of our current review.

However, 34.1% of the publishing journals belonged to the first quartile (Q1) in the hierarchy of journal's scientific impact according to Scopus®, whereas 22.0% of the included papers were published in non-indexed and unclassified journals in the main two bibliographic databases.

Orthodontic retraction of incisors, canines, or the upper six teeth together was the most applied orthodontic procedure in the included RCTs (68 trials: 74.7%). Regarding the invasiveness of the included interventions, the majority of the included trials involved minimally invasive surgical interventions (60 RCTs: 65.9%). Concerning the adherence to reporting of harms, 37 trials (40.7%) included information about the occurrence or absence of harms during the trial course. Six of them (6/37 RCTs: 16.21%) declared that harms had actually occurred with details about these events, whereas the rest (31/37 trials: 83.78%) reported the absence of any harms. On the other hand, 54 trials (59.3% of the whole included RCTs) did not report this element. Among the studies that reported harms, 18 trials (48.64%) allocated a specific paragraph under a definite subheading in the Results section, whereas in the rest of the papers (i.e., 19 trials: 51.35%), the information about possible harms or injuries were given inexplicitly in the context of the Results or Discussion sections of the manuscript. More details about the characteristics of included RCTs can be found in Table 1.

**Table 1:** Characteristics of the included 91 RCTs

Characteristic	Category	*n*	%
Year of publication	2007	1	1.1
	2011	1	1.1
	2012	2	2.2
	2013	1	1.1
	2014	3	3.3
	2015	3	3.3
	2016	10	11.0
	2017	4	4.4
	2018	11	12.1
	2019	17	18.7
	2020	17	18.7
	2021	11	12.1
	2022	10	11.0
Country of the research team	USA	3	3.3
	Australia	1	1.1
	Belgium	3	3.3
	Brazil	2	2.2
	China	3	3.3
	Colombia	1	1.1
	Cyprus	1	1.1
	Egypt	18	19.8
	India	23	25.3
	Iran	4	4.4
	Jordan	1	1.1
	Malaysia	4	4.4
	Saudi Arabia	3	3.3
	Switzerland	1	1.1
	Syria	15	16.5
	Thailand	1	1.1
	Turkey	7	7.7
HDI of the country of the research team	Very high human development	23	25.3
	High human development	12	13.2
	Medium human development	56	61.5
Journal of publication	AL Azhar Dental Journal for girls	4	4.4
	American Journal of Orthodontics and Dentofacial Orthopedics	9	9.9
	Angle Orthodontist	8	8.8
	APOS Trends in Orthodontics	1	1.1
	BMC Oral Health	3	3.3
	Clinical Oral Investigations	1	1.1
	Dental and Medical Problems	2	2.2
	Dental Research Journal	1	1.1
	Egyptian Dental Journal	2	2.2
	Egyptian Orthodontic Journal	4	4.4
	European Journal of Orthodontics	5	5.5
	Head And Face Medicine	1	1.1
	Indian Journal of Public Health Research and Development	1	1.1
	International Arab Journal of Dentistry	1	1.1
	International journal of odontostomatology	1	1.1
	International Journal of Oral and Maxillofacial Surgery	1	1.1
	International Journal Of Periodontics And Restorative Dentistry	2	2.2
	International Journal of Research in Medical Sciences	1	1.1
	International Orthodontics	2	2.2
	Journal of Advanced Medical and Dental Sciences Research	1	1.1
	Journal of American Science	1	1.1
	Journal of Clinical and Diagnostic Research	2	2.2
	Journal of Clinical and Experimental Dentistry	1	1.1
	Journal of Dental Research	1	1.1
	Journal of Indian Society of Periodontology	2	2.2
	Journal of International Academy of Periodontology	1	1.1
	Journal of International Society of Preventive and Community Dentistry	1	1.1
	Journal of Lasers in Medical Sciences	1	1.1
	Journal of Oral and Maxillofacial Surgery	2	2.2
	Journal of Oral Biology and Craniofacial Research	2	2.2
	Journal of Orhodontic Science	1	1.1
	Journal of Orofacial Orthopedics	2	2.2
	Journal of Orthodontic Science	1	1.1
	Journal of Orthodontics	1	1.1
	Journal of the International Academy of Periodontology	1	1.1
	Journal Of The International Clinical Dental Research Organization	1	1.1
	Journal of the World Federation of Orthodontists	2	2.2
	Korean Journal Of Orthodontics	1	1.1
	Laser Therapy	1	1.1
	Medical Journal Armed Forces India	1	1.1
	Medicine & Pharmacy Reports	1	1.1
	Open Access Macedonian Journal of Medical Sciences	2	2.2
	Orthodontic Waves	1	1.1
	Orthodontics And Craniofacial Research	1	1.1
	Pakistan Oral & Dental Journal	1	1.1
	Progress in Orthodontics	3	3.3
	Saudi Dental Journal	1	1.1
	The Journal of Contemporary Dental Practice	1	1.1
	The Journal of Craniofacial Surgery	1	1.1
	The Scientific World Journal	1	1.1
	Turkish Journal of Orthodontics	1	1.1
Quartile of the publishing journal	Q1	31	34.1
	Q2	22	24.2
	Q3	14	15.4
	Q4	4	4.4
	Unclassified	20	22.0
Type of orthodontic tooth movement	Retraction	68	74.7
	Decrowding	20	22.0
	various other procedures	3	3.3
Invasiveness of SAAO	Invasive	23	25.3
	Minimally invasive	60	65.9
	Both	8	8.8
Adherence to reporting harms	Yes	37	40.7
	No	54	59.3
The way of reporting harms	Reported under a definite subheading	18	19.8
	Reported inexplicitly within the Results or Discussion sections	19	20.9
	Not reported at all	54	59.3

### Risk of bias of the included studies

Of the 91 included RCTs, only 2 studies were judged as 'low risk of bias'. On the other hand, 55 RCTs were assessed as having 'some concern of bias', while the other 34 RCTs were at 'high risk of bias' (Table 2). However, the domain of deviations from intended interventions (effect of assignment to intervention or effect of adhering to intervention) was the most doubtful. The risk of bias of the included RCTs is presented in Additional file 3: Fig. S1. Moreover, the overall risk of bias for each domain is shown in Additional file 4: Fig. S2. More details about the bias risk assessment with supporting reasons for every judgment can be found in Additional file 5: Table S3.

**Table 2:** SAAO-related harms reported in the included studies

Study	Setting	Publication journal		Methods		Participants	Orthodontic procedure	Invasiveness of the SAAO	Reported harm
		Name	Quartile	Study design	Treatment comparison	Patients (M/F) Age (years)			
Al-Ainawi et al. [49]	Syria	Journal of Contemporary Dental Practice	Q3*	RCT, SMD	DAD vs. Modified DAD	Patients (M/F): 7 (NR\ NR) Age (years): 16–25	Maxillary canine retraction	Invasive	Non-vitality of the retracted canine
Tunçer et al. [47]	Turkey	European Journal of Orthodontics	Q1*	RCT, 2-Arms PG	Piezocision vs. NAC	Patients (M/F): 30 (4\ 26) Age (years): 14.3–25.5	En-mass retraction	Minimally invasive	Ectopic bony growths
Kundi and Shaheed [42]	Saudi Arabia	Pakistan Oral & Dental Journal	Unclassified**	RCT, SMD	MOPs vs. NAC	Patients (M/F): 30 (12\ 18) Age (years): 20–36	Maxillary canine retraction	Minimally invasive	Minor bleeding in the perforation area
Thind et al. [53]	India	Journal of Indian Society of Periodontology	Q4*	RCT, 2-Arms PG	PAOO with surgical bur vs. PAOO with piezocision	Patients (M/F): 40 (NR\ NR) Age (years): 20–40	Retraction	Invasive	Swelling, and hematoma of the chin for 2–3 days
Al-Imam et al. [2]	Syria	Dental and Medical Problems	Q3*	RCT, 2-Arms PG	Piezocision vs. NAC	Patients (M/F): 42 (11\ 31) Age (years): 16–31	Maxillary incisors retraction	Minimally invasive	The recession of the interdental papilla in the midline following acute inflammatory response in that area
Fernandes et al. [48]	Brazil	Progress in Orthodontics	Q1*	RCT, COMP	(Corticotomy\NAC) vs. (piezocision \NAC) vs. (Corticotomy \ piezocision	Patients (M/F): 47 (19\ 28) Age (years): 15–38	Maxillary canine retraction	Combination of IP and MIP in PG or SMDs	Bone sequestration associated with piezocision

*SAAO* surgically assisted accelerated orthodontics, *RCT* Randomized clinical trial, *SMD* Split-mouth design, *PG* Parallel group, *COMP* Compound design (it consists of both parallel and split-mouth), *DAD* Dentoalveolar distraction, *NAC* Non-Accelerated Control, *MOPs* Micro-osteoperforations, *PAOO* Periodontally accelerated osteogenic orthodontics, *NR* Not reported, *IP* Invasive procedure, *MIP* Minimally invasive procedure, *PG* Parallel group, *SMDs* Split mouth designs

^*^Indexed according to Scopus® database

^**^Not indexed by both Scopus® and Web of Science™ databases

### The possible relationship between harm reporting (HR) and the other factors

No statistically significant difference between the RCTs who reported harm and those that did not report it, with regard to the year of article publication (*U* = 845, *p* = 0.210, Table 3). On the contrary, a positive correlation was found between HR and HDI, but this correlation was weak (*r*_s_ = 0.30, *p* = 0.003, Table 3). In addition, examining the association between the HR and the CSBQ also showed a positive but weak significant correlation (*r*_s_ = 0.34, *n* = 91, *p* = 0.001, Table 3).

**Table 3:** Descriptive statistics of the proportions of studies that reported harms according to the HDI of the country of the research team, the prestige of the publishing journal, and the invasiveness of the surgical procedure along with the *p* values of statistical testing

		Reporting of Harms		Total	*P* Value
		Yes	No		
*Human Development Index of the country of the research team*					
Very high HDI	*n*	14	9	23	< 0.05^a^
	%	15.4%	9.9%	25.3%	
High HDI	*n*	7	5	12	
	%	7.7%	5.5%	13.2%	
Medium HDI	*n*	16	40	56	
	%	17.6%	44.0%	61.5%	
*Quartile of the publishing journal*					
Q1	*n*	17	14	31	< 0.05^a^
	%	18.7%	15.4%	34.1%	
Q2	*n*	12	10	22	
	%	13.2%	11.0%	24.2%	
Q3	*n*	5	9	14	
	%	5.5%	9.9%	15.4%	
Q4	*n*	1	3	4	
	%	1.1%	3.3%	4.4%	
Not indexed	*n*	2	18	20	
	%	2.2%	19.8%	22.0%	
*Invasiveness of the SAAO*					
Invasive	*n*	4	19	22	< 0.05^b^
	%	4.4%	20.9%	24.2%	
Minimally invasive	*n*	30	30	61	
	%	33.0%	33.0%	67.0%	
Both	*n*	3	5	8	
	%	3.3%	5.5%	8.8%	
*Type of orthodontic tooth movement*					
Retraction	*n*	26	42	68	> 0.05^b^
	%	28.6%	46.2%	74.7%	
Decrowding	*n*	10	10	20	
	%	11.0%	11.0%	22.0%	
All other OTM	*n*	1	2	3	
	%	1.1%	2.2%	3.3%	
Total	*n*	37	54	91	
	%	40.7%	59.3%	100.0%	

*SAAO* surgically assisted accelerated orthodontics, *HDI* Human Development Index, *Q* Quartile to which the journal belongs, *OTM* Orthodontic tooth movements

^a^Spearman's Rho test

^b^Fisher's exact test

**Fig. 3:**
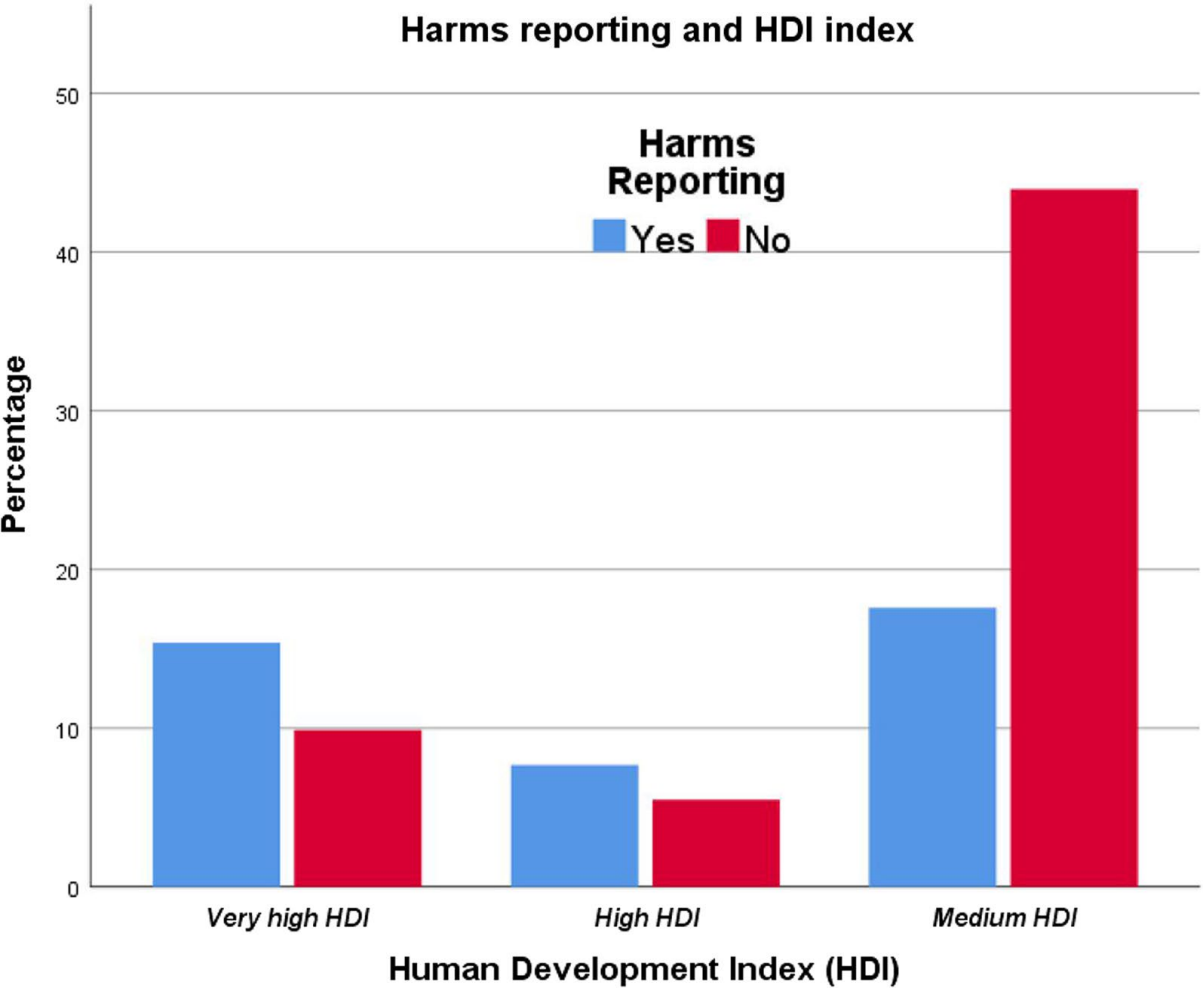
The proportions of papers reporting harms according to the HDI of the research team country

**Fig. 4:**
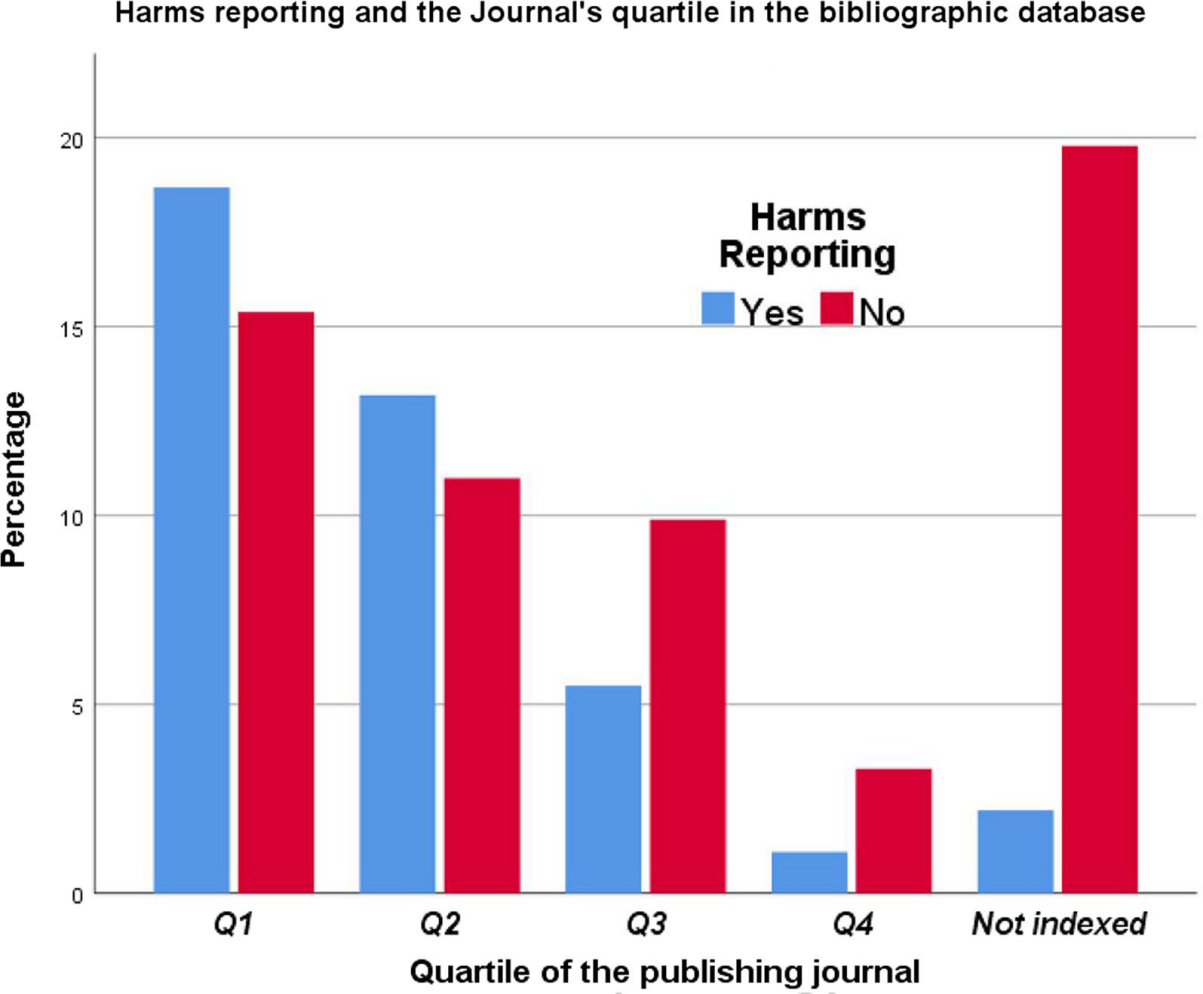
The proportions of papers reporting harms according to the database-related quartile of the publishing journal

**Fig. 5:**
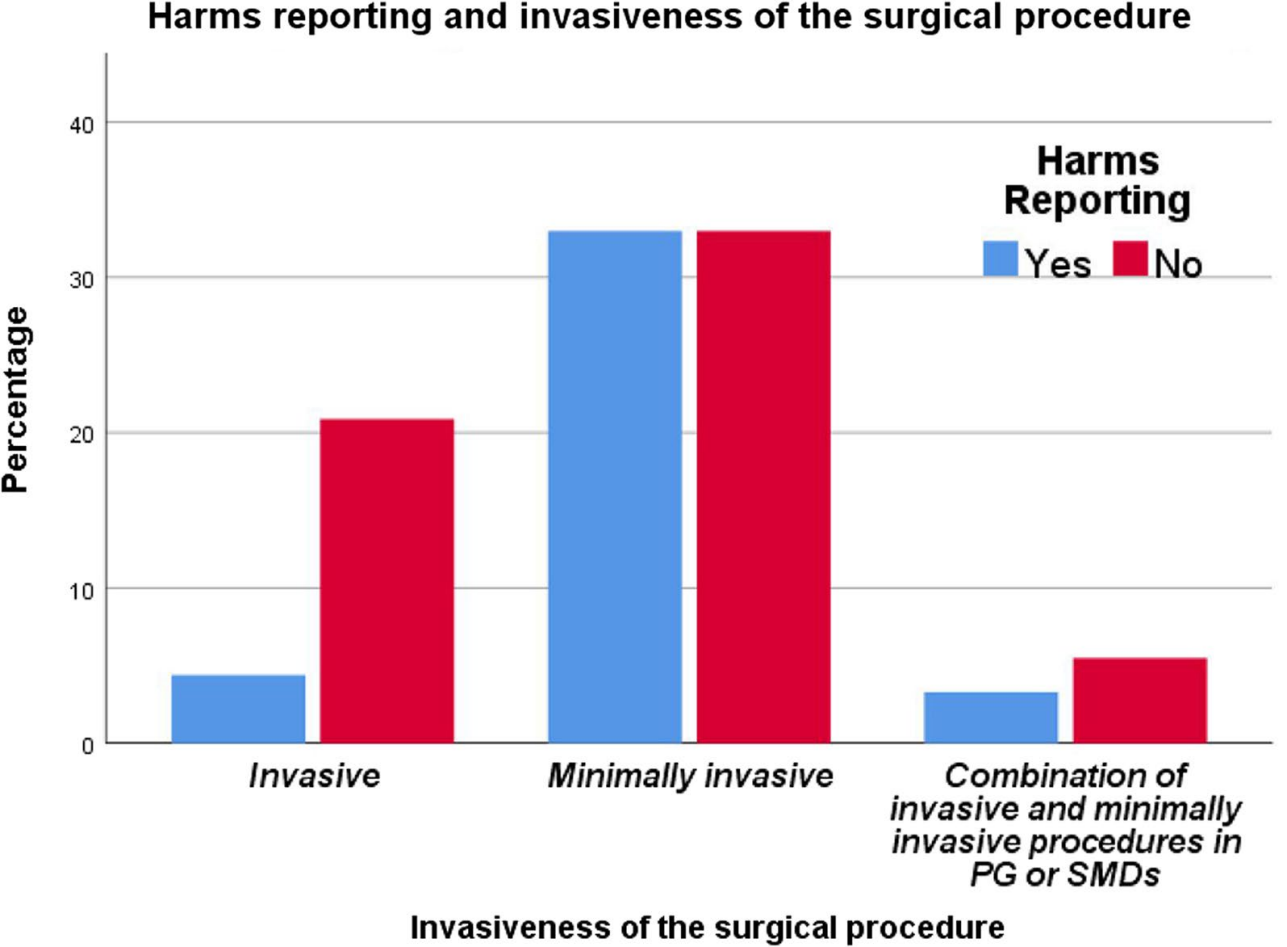
The proportions of papers reporting harms according to the invasiveness of the surgical interventions

**Fig. 6:**
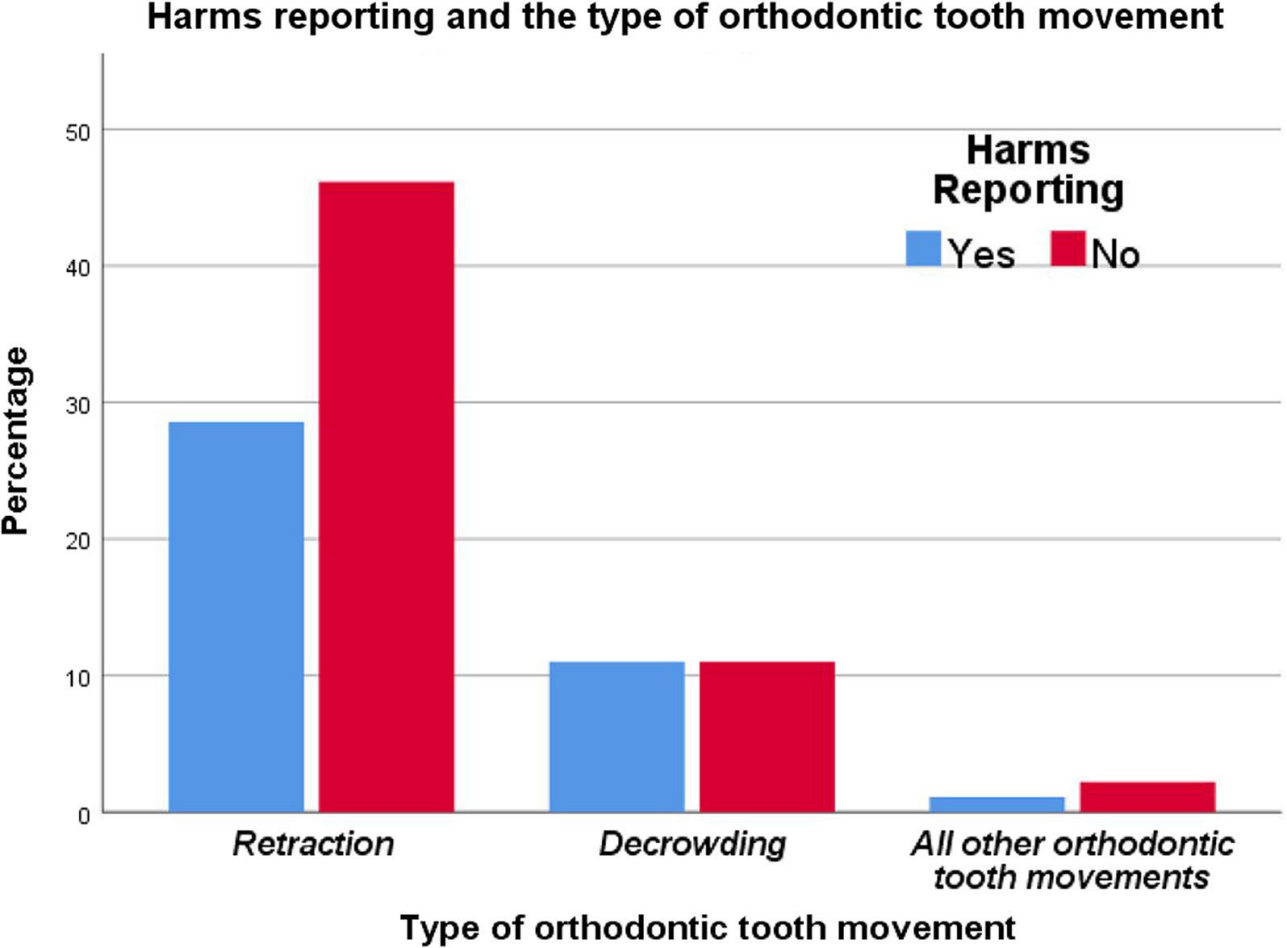
The proportions of papers reporting harms according to the type of orthodontic tooth movement

An association between HR and the invasiveness of SAAO was tested. However, the result presented a moderate, positive significant association between the two variables (*x*^2^ = 7.543, *φ*_c_ = 0.28; *p* = 0.025, Table 3). The finding of the association between HR and the type of OTM showed a non-significant association between the two variables (*p* = 0.695). More information about the descriptive statistics of the proportions and the association between HR and other variables can be found in Table 3 and Figs. 3, 4, 5 and 6.

A binary logistic regression was carried out to assess the effect of HDI, the journal's quartile, and the invasiveness of the SAAO on the likelihood of reporting SAAO-related harms. The overall model was statistically significant when compared to the null model, (*χ*^2^(8) = 23.731, *p* = 0.003), which explained 31% of the variation of harms reporting (Nagelkerke R^2^) and correctly predicted 71.4% of cases. The Hosmer and Lemeshow goodness-of-fit test showed that this model had a good fit (*p* > 0.05). However, the odds of non-adherence with the harm reporting in invasive surgical methods was 0.16 times the odds of non-adherence in minimally invasive surgical methods (95% confidence interval [CI], 0.03–0.73; *P* < 0.05; Table 4).

**Table 4:** Distribution of harms reporting (Yes/No) across other variables and the results of binary logistic regression modeling

Factors	Harms reporting		β	OR [95% CI]^¶^	*P* value^¶^
	Yes, *n* (%)	No, *n* (%)			
*HDI*					
Very high HDI	14 (37.8)	9 (16.7)		Reference	
High HDI	7 (18.9)	5 (9.3)	− 0.132	0.87 [0.18–4.25]	0.870
Medium HDI	16 (43.2)	40 (74.1)	0.928	2.52 [0.60–10.49]	0.201
*Journal's Quartile*					
Q1	17 (45.9)	14 (25.9)		Reference	
Q2	12 (32.4)	10 (18.5)	− 0.721	0.48 [0.12–1.92]	0.304
Q3	5 (13.5)	9 (16.7)	0.042	1.04 [0.18–5.82]	0.962
Q4	1 (2.7)	3 (5.6)	− 1.049	0.35 [0.01–6.99]	0.492
Not indexed	2 (5.4)	18 (33.3)	1.625	5.07 [0.79–32.43]	0.086
*Invasiveness of the SAAO*					
Invasive	4 (10.8)	19 (35.2)		Reference	
Minimally invasive	30 (81.1)	30 (55.6)	− 1.799	0.16 [0.03–0.73]	0.018*
Combination of IP and MIP in PG or SMDs	3 (8.1)	5 (9.3)	− 1.700	0.18 [0.01–1.76]	0.142

*OR* Odds ratio, *CI* Confidence interval, *HDI* Human Development Index, *Q* Quartile, *SAAO* Surgically assisted accelerated orthodontics, *IP* Invasive procedure, *MIP* Minimally invasive procedure, *PG* Parallel group, *SMDs* Split mouth designs

^¶^Binary logistic regression results

^*^Significant at the level of 0.05

### Responses from authors regarding SAAO-related harms

Of the included RCTs, 54 studies did not adhere to reporting harm. When trying to communicate with authors via email, there were 8 corresponding authors whose email was not reported in the article. While the mentioned email address of one author was no longer available. In the end, 45 emails were sent to the corresponding authors asking them about the harms associated with SAAO in their RCTs. In the beginning, 17/45 authors responded to our mail. Later a reminder email was sent to authors who did not respond. As a result, we received a response from 6 other authors. However, 13 authors [28–40] reported that SAAO-related harms were observed in their trials. On the contrary, we received a report from 10 authors of the SAAO-related harms that occurred in their trials.

### SAAO-related harms reported in the included trials

Of the 37 RCTs that reported a statement about any possible harms, 6 RCTs declared the presence of harms along with relevant details. From the 54 RCTs that did not address this issue, the corresponding authors of these papers were contacted and 10 trials were found associated with harms as shown in Tables 2 and 5 and Additional file 6: Table S4.

**Table 5:** Distribution and proportions of the recorded harms during the SAAO in the included papers along with the category of harm, the causative surgical intervention, and the treatment provided if mentioned

Harms category				Surgical intervention	Additional information
Gingival soft tissue harms	Gingival recession	*n*	1	Piezocision [2]	The patient was given the necessary care, then was excluded from the study because he neglected oral hygiene instructions, which caused this complication [2]
		%	1.1%		
	Gingival bleeding or tearing	*n*	4	MOPs [41–44]	The bleeding was minor [41, 42] The hemostasis was achieved by simple pressure application [43] Full recovery from gingival tearing took about two weeks [44]
			4.4%		
	Infection	*n*	2	Corticotomy [46]	The harm was managed by ordaining antibiotics for a week, and painkillers for 4 days [46]
		%	2.2%	Piezocision [45]	The abscess receded within a week with antibiotics, analgesics, and oral rinsing agents [45]
	Scarring	*n*	1	Piezocision, LAFC [45]	The two cases were asked to wait, then after about 8 months of follow-up, a spontaneous improvement was noted and only slight traces of scars remained [45]
		%	1.1		
	Total	*n*	7		
		%	7.7%		
Alveolar bone harms	Ectopic bony growths	*n*	1	Piezocision [47]	7 out of 15 patients suffering from this harm with no more information about this problem or how to solve it, except for an attached image that demonstrated this phenomenon [47]
		%	1.1%		
	Bone sequestration	*n*	1	Piezocision [48]	The harm was solved without major sequelae [48]
		%	1.1%		
	Total	*n*	2		
		%	2.2%		
Dental harms	Tooth vitality loss	*n*	1	DAD [49]	3 out of 7 U3 (42,86%) were nonvital in the groups of traditional DAD and modified DAD, without any discoloration or pulpal pain in any of the distracted U3 [49]
		%	1.1%		
	Tooth sensitivity	*n*	1	FTMPF [50]	The sensitivity lasted for 5 days [50]
		%	1.1%		
	Total	*n*	2		
		%	2.2%		
PROMs related harms	Pain	*n*	3	MOPs [41, 51], Piezocision [52]	The post-piezocision pain lasted for a few days [52]. The post-MOPs pain was mild [51]
		%	3.3%		
	Discomfort	*n*	2	FTMPF [50], MOPs [43]	The post-FTMPF discomfort lasted for 2 days and then disappeared [50]
		%	2.2%		
	Swelling	*n*	5	PAOO [53], FTMPF [50], Piezocision [52], MOPs [44, 51]	3 out of 20 patients in the PAOO with piezocision group experienced swelling [53] The post-FTMPF swelling lasted for 2 days [50] The post-piezocision swelling lasted for a few days [52] The swelling was during the first post-MOPs week [44]
		%	5.5%		
	Psychological harms	*n*	2	Corticision [54], MOPs [41]	Because fear of undergoing surgical intervention, one patient suffered from dizziness and hypotension. The case was managed and the patient was monitored until she returned to her normal condition. Later, she was contacted on the same day and confirmed that she was in good health without any symptoms [54] The monthly repetition of MOPs caused fear since some patients were afraid and asked to not undergo the perforations [41]
		%	2.2%		
	Total	*n*	8		
		%	8.8%		
Other harms	Numbness	*n*	1	Corticotomy [46]	One patient had numbness in the corner of the upper lip, which lasted for approximately a month and then disappeared after being given nerve repair medication [46]
		%	1.1%		
	Hematoma	*n*	2	PAOO [53], Flapless corticotomy using bur [55]	3 out of 20 patients in the PAOO with piezocision group experienced hematoma of the chin [53] One patient developed a significant hematoma in the lower lip while applying flapless corticotomy using bur [55]
		%	2.2%		
	Total	*n*	3		
		% of total	3.3%		
Total	*n*	16			
	% of total	17.6%			

*SAAO* surgically assisted accelerated orthodontics, *MOPs* Micro-osteoperforations, *LAFC* Laser-assisted flapless corticotomy, *DAD* Dentoalveolar distraction, *U3* Upper canine, *FTMPF* full-thickness mucoperiosteal flap, *PAOO* Periodontally accelerated osteogenic orthodontics

Gingival harms were documented in 7 (7.7%) papers. These included gingival recession [2], gingival bleeding [41–43], tearing of the gingival tissues [44], localized infection [45, 46], and gingival scarring following healing [45]. Alveolar bone harms were found in two trials (2.2%; Table 5). These harms were either ectopic bony overgrowths [47] or bone sequestration [48]. On the other hand, dental harms were found in two papers (2.2%; Table 5) and these were loss of tooth vitality [49] and tooth sensitivity [50]. Harms that were related to patient-centered outcomes were mentioned in eight papers (8.8%, Table 5). These complications had several forms such as postoperative pain [41, 51, 52], discomfort [43, 50], swelling (which was reported by the patients themselves and not those observed by the researchers) [44, 50–53], and in one paper, dizziness and hypotension accompanied by fear of undergoing the surgical intervention [41]. Also, there was a fear of repeating the surgical intervention [54]. On the other hand, numbness [46] and hematoma [53, 55] were other harms associated with SAAO, with a proportion of 3.3% (i.e., 3/92 trials; Table 5). However, in all the harms that were reported, no validated tool was used to measure the occurred harm, only the type of it was mentioned.

## Discussion

In recent years, the trend toward the application of OTM acceleration methods, especially surgical ones, has increased. Undoubtedly, any intervention carries potential risks. Therefore, before making any treatment decision, it is necessary to weigh the potential benefits and risks in order to provide the best possible treatment for the patient [56]. This leads to the question about the frequency and intensity of the possible associated harms with the SAAO and whether such harms have been adequately reported in the RCTs published in this regard.

The number of English RCTs carried out in the field of surgically accelerating OTM clearly increased, particularly in the last 5 years. The increasing desire of orthodontists to reduce the duration of orthodontic treatment to fulfill the requirements of adult patients [56], maybe one of the reasons behind the increasing research conducted in the field of SAAO.

Of the included RCTs, the percentage of non-adherence to reporting harms has reached 59.3%. This result is not surprising, as a low level of HR adherence is well recognized across other fields of dentistry [57, 58]. In addition, many studies in the medical field showed that adherence to CONSORT guidelines including the harms item was suboptimal and need to improve [59–61]. However, poor reporting of harm can be explained by some reasons. One of them may be the authors' lack of awareness of the details of harms-related data reporting accompanying the trials, which were clarified in the paper of the CONSORT extension to harms [15]. On the contrary, some researchers prefer to focus on the positive aspects of the intervention, so they omit the occurred harms [62] which may be also one of the reasons for the underreporting of harm. Another reason could be publication bias, where researchers may believe that reporting harms associated with their research may negatively affect the ability to publish their trials, may revoke approval for funding for their research work [61, 62], or may affect negatively the widespread of their proposed surgical intervention [63]. On the other hand, clinical trial participants need to be clearly and adequately informed of both the potential risks and benefits of the proposed intervention [64]. Consequently, patients who have not received an adequate explanation of the nature of potential harms following surgical interventions may not be able to adequately report them.

According to our findings, there was no significant difference between the RCTs that reported harm and those that did not, with regard to the year of article publication. This result is in agreement with the findings of Khan et al. study [65], which found that HR in RCTs published in 3 high-impact cardiovascular journals, did not improve significantly over the study period (2011–2017).

Regarding the HDI, a positive correlation between the HR and the HDI was found. This means RCTs conducted in developed countries are more transparent in reporting SAAO-related harms. However, this contrasts with the results of Contopoulos-Ioannidis et al., who reported in their MES that poor reporting of harms is a global problem regardless of where the RCTs were conducted [20].

Concerning the CSBQ of the publishing journal, the correlation between HR and the CSBQ was positive. However, many high-quartile journals request authors to present the CONSORT checklist with manuscript submissions [66], which could be the reason behind the increased commitment of HR in the trials published in these journals. Although the correlation between the HR and both HDI and the CSBQ was statistically significant, it was a weak correlation. Therefore, this weak correlation is clinically insignificant for orthodontic practitioners.

Minimally invasive procedures (MIPs) dominated the acceleration techniques applied in the included studies. However, MIPs are flapless, therefore the pain and discomfort associated with these techniques are minimal, which positively affects the patient's acceptance [67]. In addition, recovery is relatively faster, and the procedure time is less compared to other invasive procedures. This may be the reason for the popularity that MIPs have gained.

On the other hand, the odds ratio of not reporting harms in RCTs that applied invasive surgical acceleration methods was 0.16 times higher than in RCTs that applied minimally invasive methods. However, surgical acceleration methods, regardless of the technique applied, are fairly invasive, and therefore can be associated with complications [10]. Furthermore, the more invasive, the more likely complications will occur. As mentioned earlier, authors may resort to hiding data related to Harms for fear that it will be difficult to publish their research [62]. This may be the rationale behind the underreporting of harms in RCTs of invasive methods.

Regarding the type of OTM, no association between HR and the type of OTM applied in the included RCTs, was found. Of the included RCTs, orthodontic retraction (whether for canine, incisor, or en-mass) was the most frequently applied orthodontic procedure in the included studies. The reason for this may be that retraction cases require a total treatment period of about 2 years [68]. Therefore, acceleration procedures of OTM may be useful in these cases.

Bias defines as "systematic error, or deviation from the truth, in results" [69]. In our study, of the included RCTs, only two trials were assessed as low risk of bias, while the rest of the articles were either high risk or had some concern of bias. However, with the articles biased, the results of the trials will be affected and thus this affects the results reported in these articles, of which 'harms reporting'. This was noticed because some of the authors we contacted reported the occurrence of harms that were not mentioned in the text of the article.

Regarding the harms associated with SAAO, whether mentioned in studies or reported by authors via email, most were found to fall into the two categories of SAAO-related PROMs harms and SAAO-related gingival harms. However, concerning harms associated with invasive SAAO, DAD can be accompanied by loss of tooth vitality [49]. While PAOO may be associated with occurring swelling and hematoma [53]. On the other hand, conventional corticotomy may cause severe gingival inflammation and numbness. Whereas, full-thickness mucoperiosteal flap procedure may cause discomfort, edema, and tooth sensitivity [50]. Regarding harms associated with minimally invasive SAAO, the application of piezocision can be accompanied by ectopic bony growth [47], bone sequestration [48], gingival recession [2], infection [2, 45], scarring [45], pain, and swelling[52]. On the other hand, the application of MOPs may be associated with the following complications: bleeding [41, 43], redness [51], bruising [44], mucosal tear [44], pain [41, 51], discomfort [43], and swelling [44, 51].

The fact that SAAO-related harms occurred and did not mention in the published articles may indicate one of two things: either there is ignorance regarding the importance of item 19 of CONSORT, or this information has been deliberately hidden out of fear of refusal to accept the article’s publication.

In future trials, researchers must have an obligation to report harms that may be associated with their implementation of the surgical acceleration method. In addition, this notification must be sufficient by stating the nature of the damage, when it occurred, and how it was managed. Additionally, journal editors and reviewers should be more stringent about “harms reporting” as this has an impact on whether or not to adopt the proposed intervention.

## Limitations

Only English RCTs related to SAAO were included in this study. This study focused on the investigation of adherence to reporting harm associated with SAAO and did not address the other non-surgical acceleration methods. Moreover, other factors such as the number of medical centers involved in the RCT and the nature of funding for the RCT that may have a potential impact on the transparency of reporting harms, were not studied in this meta-epidemiological study.

## Conclusion

The proportion of adherence to reporting harms in the field of SAAO was substandard (40.7%). Authors, peer reviewers, and editors must be stricter regarding compliance with the CONSORT guidelines regarding harms reporting. This will allow to weigh the benefits and harms of surgical acceleration techniques and thus optimally adopt the best procedure with the least complications or adverse effects. Patients should be fully aware of the complications that may accompany undergoing SAAO. Likewise, researchers must be fully aware of the harms associated with SAAO. On the other hand, it should be known that some factors may play a role in the adherence to reporting harm in the field of SAAO such as the scientific strength of the publishing journal assessed by the CiteScore-based quartile, the HDI of the recruited research sample country, and the invasiveness of the surgical intervention.

## Supplementary Information

**Additional file 1: Table S1**. Electronic Search Strategy.

**Additional file 2: Table S2**. Excluded studies after reading the full text and the reasons beyond exclusion.

**Additional file 3: Fig. S1**. Risk of bias summary of the included RCTs. Low risk of bias (the + sign); some concern of bias (the - sign); high risk of bias (the X sign).

**Additional file 4: Fig. S2**. Summary of the percentages of the risk of bias assessment for each domain.

**Additional file 5: Table S3**. Details of the risk of bias assessment of the randomized controlled trials using the ROB-2 tool.

**Additional file 6: Table S4**. Responses from authors regarding the SAAO-related harms.

## Data Availability

The datasets and spreadsheets related to this manuscript can be obtained from the corresponding author upon reasonable request.

## Abbreviations

SAAO: Surgically assisted accelerated orthodontics
CONSORT: Consolidated Standards of Reporting Trials
HDI: Human development index
CSBQ: CiteScore-based quartile
ISI: Invasiveness of the surgical intervention
TOTM: Type of orthodontic tooth movement
OTM: Orthodontic tooth movement
SPIRIT: Standard Protocol Items: Recommendations for Interventional Trials
RCT: Randomized controlled trials
MES: Meta-epidemiological study
PROMs: Patient-reported outcome measures
UNDP: United Nations Development Programme
CS: CiteScore
JIF: Journal impact factor
JCI: Journal citation indicator
DAD: Dentoalveolar distraction
PAOO: Periodontally accelerated osteogenic orthodontics
MOPs: Micro-osteoperforations
LAAC: Laser-assisted flapless corticotomy
U: Mann-Whitney test
*r*_s_: Spearman's Rho test
*φ*_c_: Cramer's V
ORs: Odds ratios
HR: Harm reporting
