# Supplementary material for: The transparency of reporting 'harms' encountered with the surgically assisted acceleration of orthodontic tooth movement in the published randomized controlled trials: a meta-epidemiological study
Source: Prog Orthod. 2023 Mar 21;24:11. doi: 10.1186/s40510-023-00457-4 (PMC10027979; doi:10.1186/s40510-023-00457-4)
Supplement: Supplementary file 1 — Additional file 1: Table S1. Electronic Search Strategy. [file 40510_2023_457_MOESM1_ESM.docx]

| **Supplementary Table 1:** Electronic Search Strategy | |
| --- | --- |
| **Database** | **Search Strategy** |
| **CENTRAL (The Cochrane Library)**  **Publication Date: from January 2000 Until April 2022 Search field:  title ,abstract,**  **keywords** | #1 orthodontic* OR "Tooth movement" OR "orthodontic tooth movement” OR "orthodontic Treatment" OR "orthodontic Therapy"  #2 accelerat* OR rapid* OR short* OR speed* OR fast OR velocity OR duration OR rate OR time OR "regional accelerated phenomenon" OR RAP.  #3 (surgical( AND (corticotom* OR decorticat* OR alveolar surg* OR piezosurgery OR piezoelectric OR piezo* OR microsurgery OR micro incisions OR micro osteoperforations OR micro-perforations OR perforations OR corticision OR lasercision OR corticopuncture OR piezocision OR piezotome OR piezopuncture)  #4 #2 OR #3 OR  #5 #1 AND #4 |
| **PubMed**  **Publication Date: from January 2000 Until April 2022** | (orthodontics [Mesh] OR "Tooth movement" OR "orthodontic tooth movement” OR "Tooth displacement “OR "orthodontic Treatment" OR "orthodontic Therapy") AND (accelerate OR acceleration [Mesh] OR accelerating OR accelerated OR rapid) AND (corticotom* OR decorticat* OR alveolar surg* OR piezosurgery [Mesh] OR piezoelectric OR piezo* OR microsurgery OR micro incisions OR micro osteoperforations OR micro-perforations OR perforations OR corticision OR lasercision OR corticopuncture OR piezocision OR piezotome OR piezopuncture) |
| **Scopus**  **Publication Date:**  **from January 2000 Until April 2022** | #1TITLE-ABS-KEY (orthodontic* OR "Tooth movement" OR "orthodontic tooth movement” OR "orthodontic Treatment" OR "orthodontic Therapy").  #2TITLE-ABS-KEY (accelerat* OR rapid* OR short* OR speed* OR fast OR velocity OR duration OR rate OR time OR "regional accelerated phenomenon" OR RAP).  #3 TITLE-ABS-KEY ("surgical") AND TITLE-ABS-KEY (corticotom* OR decorticat* OR alveolar surg* OR piezosurgery OR piezoelectric OR piezo* OR microsurgery OR micro incisions OR micro osteoperforations OR micro-perforations OR perforations OR corticision OR lasercision OR corticopuncture OR piezocision OR piezotome OR piezopuncture).  #4 #2 OR #3 OR  #5 #1 AND #4 |
| **Web of Science**  **Publication Date:**  **from January 2000 Until April 2022** | #1TS= (orthodontic OR "Tooth movement" OR "orthodontic tooth movement” OR "Tooth displacement “OR "orthodontic Treatment" OR "orthodontic Therapy").  #2TS= (accelerat* OR rapid* OR short* OR speed* OR fast OR velocity OR duration OR rate OR time OR "regional accelerated phenomenon" OR RAP).  #3TS= (surgical) AND TS= (corticotom* OR decorticat* OR alveolar surg* OR piezosurgery OR piezoelectric OR piezo* OR microsurgery OR micro incisions OR micro osteoperforations OR micro perforations OR perforations OR corticision OR lasercision OR corticopuncture OR piezocision OR piezotome OR piezopuncture).  #4 #2 OR #3 OR  #5 #1 AND #4 |
| **Google Scholar**  **Publication Date:**  **from January 2000 Until April 2022** | #1 (orthodontic OR "Tooth movement" OR "orthodontic tooth movement” OR "Tooth displacement “OR "orthodontic Treatment" OR "orthodontic Therapy") AND (accelerate OR acceleration OR accelerating OR accelerated OR rapid) AND (surgical)  #2 (orthodontic OR "Tooth movement" OR "orthodontic tooth movement” OR "Tooth displacement “OR "orthodontic Treatment" OR "orthodontic Therapy") AND (accelerate OR acceleration OR accelerating OR accelerated OR rapid) AND (corticotom* OR decorticat* OR alveolar surg* OR piezosurgery OR piezoelectric OR piezo* OR microsurgery OR micro incisions OR micro osteoperforations OR micro-perforations OR perforations OR corticision OR lasercision OR corticopuncture OR piezocision OR piezotome OR piezopuncture) |
| **OpenGrey**  **http://www.opengrey.eu/** | Orthodontic |
