# Supplementary material for: The transparency of reporting 'harms' encountered with the surgically assisted acceleration of orthodontic tooth movement in the published randomized controlled trials: a meta-epidemiological study
Source: Prog Orthod. 2023 Mar 21;24:11. doi: 10.1186/s40510-023-00457-4 (PMC10027979; doi:10.1186/s40510-023-00457-4)
Supplement: Supplementary file 2 — Additional file 2: Table S2. Excluded studies after reading the full text and the reasons beyond exclusion. [file 40510_2023_457_MOESM2_ESM.docx]

| **Supplementary Table 2:** Excluded studies after reading the full text and the reasons beyond exclusion | |
| --- | --- |
| **Study** | **Reason for exclusion** |
| Ahmed, O. E., El Kilani, N. S., Ibrahim, S. A., Salama, A. E., & Khalifa, G. A. (2020). Clinical and Radiographic Evaluation of Piezocision Corticotomy with Bone Graft Guided By 3D-Surgical Template in Maxillary Protrusion (comparative study). Al-Azhar Dent J Girls, 447-451.‏ | Non-RCT |
| Karacay, S., Saygun, I., Bengi, A. O., & Serdar, M. (2007). Tumor Necrosis Factor–α Levels during Two Different Canine Distalization Techniques. Angle Orthod, 77(1), 142-147.‏ | Non-RCT |
| Suryavanshi, H., Das, V., Deshmukh, A., Rai, R., & Vora, M. (2015). Comparison of rate of maxillary canine movement with or without modified corticotomy facilitated orthodontic treatment: A prospective clinical trial. APOS Trends Orthod, 5(4), 138-138.‏ | Non-RCT |
| Chawshli, O., Omer, Z., & Ikram, O. (2018). Accelerated Orthodontic Canine Retraction Using Minimally Invasive Orthocision. EDJ, 1(1), 19-25.‏ | Non-RCT |
| Kateel, S. K., Agarwal, A., Kharae, G., Nautiyal, V. P., Jyoti, A., & Prasad, P. N. (2016). A comparative study of canine retraction by distraction of the periodontal ligament and dentoalveolar distraction methods. J Oral Maxillofac Surg, 15(2), 144-155.‏ | Non-RCT |
| Elkalza, A. R. (2017). Evaluation of piezocision in rapid canine retraction. Egypt Dent J, 51(June 2017), 59-71.‏ | Non-RCT |
| Strippoli, J., Durand, R., Schmittbuhl, M., Rompré, P., Voyer, R., Chandad, F., & Nishio, C. (2019). Piezocorticision-assisted orthodontics: Efficiency, safety, and long-term evaluation of the inflammatory process. Am J Orthod Dentofacial Orthop, 155(5), 662-669.‏ | Non-RCT |
| Mezari, A., & Ahmed, F. S. (2018). Study of the velocity of upper canine retraction after alveolar corticotomy. JDAO, 21(4), 507.‏ | Non-RCT |
| Uzuner, F. D., Yücel, E., Göfteci, B., & Gülsen, A. (2015). The effect of corticotomy on tooth movements during canine retraction. J Orthod Res, 3(3), 181.‏ | Non-RCT |
| Yashwant V, A., Balu, P., Kumar, R. S., Ammayappan, P., & Murugaboopathy, V. (2022). Effectiveness of platelet rich fibrin versus demineralized bone xenograft in periodontally accelerated osteogenic orthodontics: A pilot comparative clinical study. Angle Orthod, 92(2), 180-188.‏ | Non-RCT |
| Non-RCT: Non-Randomized controlled trial | |
