# Supplementary figures and images for: The transparency of reporting 'harms' encountered with the surgically assisted acceleration of orthodontic tooth movement in the published randomized controlled trials: a meta-epidemiological study
Source: Prog Orthod. 2023 Mar 21;24:11. doi: 10.1186/s40510-023-00457-4 (PMC10027979; doi:10.1186/s40510-023-00457-4)

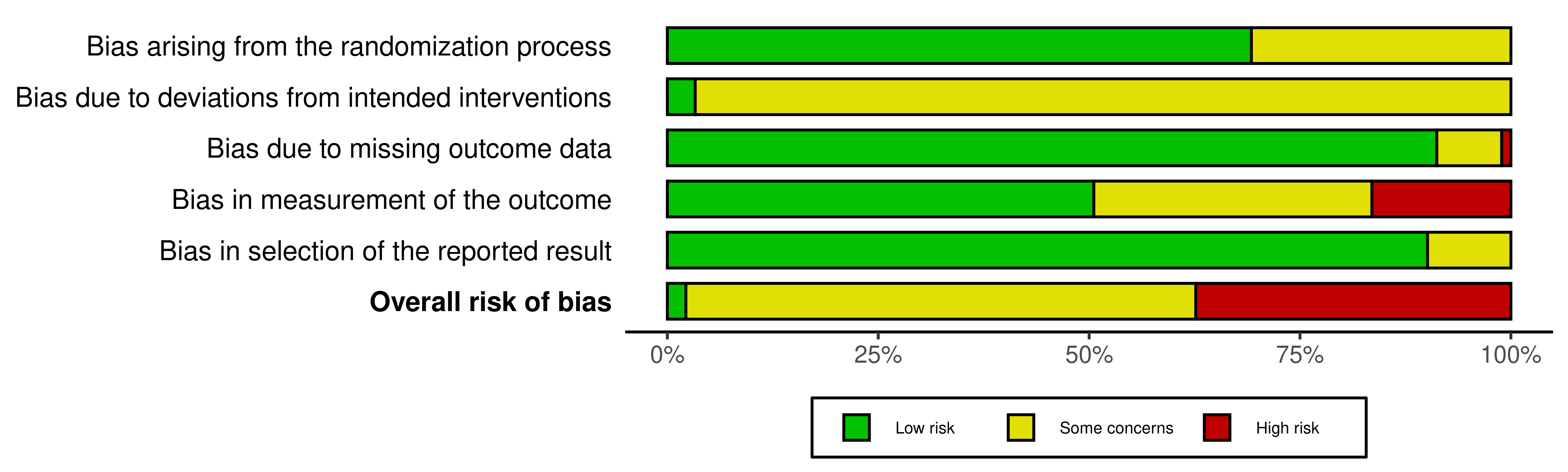

Supplement: Supplementary file 4 — Additional file 4: Fig. S2. Summary of the percentages of the risk of bias assessment for each domain. [file 40510_2023_457_MOESM4_ESM.png]
