# Supplementary material for: The transparency of reporting 'harms' encountered with the surgically assisted acceleration of orthodontic tooth movement in the published randomized controlled trials: a meta-epidemiological study
Source: Prog Orthod. 2023 Mar 21;24:11. doi: 10.1186/s40510-023-00457-4 (PMC10027979; doi:10.1186/s40510-023-00457-4)
Supplement: Supplementary file 5 — Additional file 5: Table S3. Details of the risk of bias assessment of the randomized controlled trials using the ROB-2 tool. [file 40510_2023_457_MOESM5_ESM.docx]

| **Supplementary Table 3:** Details of the risk of bias assessment of the randomized controlled trials according to the ROB-2 tool | | | | | | | |
| --- | --- | --- | --- | --- | --- | --- | --- |
| **Study** | **Bias arising from the randomization process** | **Bias due to deviations from intended interventions** | | **Bias due to missing outcome data** | **Bias in measurement of the outcome** | **Bias in selection of the reported result** | **Overall bais** |
|  |  | **effect of assignment to intervention** | **effect of adhering to intervention** |  |  |  |  |
|  | **D1** | **D2** | | **D3** | **D4** | **D5** |  |
| **Fischer 2007** | **Some concerns:**  The method used for randomization was not reported.  "By random selection, one canine had a conventional surgical uncovering procedure. On the other canine an additional corticotomy procedure was performed” (Page 418) | **Some concerns:**  Blinding cannot be performed.  There is ‘No information’ on whether any deviations arose because of the trial context. | **Some concerns:**  Blinding cannot be performed.  And ‘No information’ on whether the important non-protocol interventions were balanced across intervention groups | **Low risk:**  No dropouts were reported. | **Some concerns:**  No details of blinding of outcome assessors were reported.  The method of measuring the outcome is appropriate. | **Low risk:**  No information about the registration protocol was mentioned. But the reported outcomes in the result section seemed to be corresponding with the pre-defined outcomes aforesaid in the method section. | **High risk** |
| **Aboul-Ela 2011** | **Low risk:**  "Corticotomy-facilitated orthodontics was randomly assigned to 1 side of the maxillary arch at the canine premolar region” (Page 252)  "On the day before the corticotomy surgery, 1 maxillary premolar was extracted on a random basis (coin toss).” (Page 253) | **Some concerns:**  Blinding cannot be performed.  There is ‘No information’ on whether any deviations arose because of the trial context. | **Some concerns:**  Blinding cannot be performed.  And ‘No information’ on whether the important non-protocol interventions were balanced across intervention groups | **Low risk:**  “2 patients were excluded from the study—1 because of multiple missed appointments and the other because of poor oral hygiene” (Page 254)  Nearly all” the outcome data is available. | **Some concerns:**  No details of blinding of outcome assessors were reported.  The method of measuring the outcome is appropriate. | **Low risk:**  No information about the registration protocol was mentioned. But the reported outcomes in the result section seemed to be corresponding with the pre-defined outcomes aforesaid in the method section. | **Some concerns** |
| **Abbas 2012** | **Some concerns:**  The method used for randomization was not reported.  "Patients were randomly divided to two groups” (Page 15) | **Some concerns:**  Blinding cannot be performed.  There is ‘No information’ on whether any deviations arose because of the trial context. | **Some concerns:**  Blinding cannot be performed.  And ‘No information’ on whether the important non-protocol interventions were balanced across intervention groups | **Low risk:**  No dropouts were reported. | **High risk:**  No details of blinding of outcome assessors and the method of measuring the outcome were reported. | **Some concerns:**  No information about the registration protocol was mentioned.  Moreover, not all outcome variables to be studied, may not have been reported as the details reported in the Methods section were insufficient. | **High risk** |
| **Shoreibah 2012** | **Some concerns:**  The method used for randomization was not reported.  "Twenty adult orthodontic patients... were randomly divided and treated” (Page 97) | **Some concerns:**  Blinding cannot be performed.  There is ‘No information’ on whether any deviations arose because of the trial context. | **Some concerns:**  Blinding cannot be performed.  And ‘No information’ on whether the important non-protocol interventions were balanced across intervention groups | **Low risk:**  ‘No dropouts were reported. | **Some concerns:**  No details of blinding of outcome assessors were reported.  The method of measuring the outcome is appropriate. | **Low risk:**  No information about the registration protocol was mentioned. But the reported outcomes in the result section seemed to be corresponding with the pre-defined outcomes aforesaid in the method section. | **High risk** |
| **Alikhani 2013** | **Some concerns:**  The method used for randomization was not reported.  "Patients who met the selection criteria and completed an informed consent form were randomly assigned to one of the study groups.” (Page 640) | **Some concerns:**  “The subjects and the residents administering the treatment were aware of the group assignment and therefore were not blinded.” (Page 640)  There is ‘No information’ on whether any deviations arose because of the trial context. | **Some concerns:**  “The subjects and the residents administering the treatment were aware of the group assignment and therefore were not blinded.” (Page 640)  And ‘No information’ on whether the important non-protocol interventions were balanced across intervention groups | **Low risk:**  "no loss to follow-up.” (Page 642) | **Low risk:**  "The investigators performing the measurements and data analysis were blinded from the group assignments.” (Page 640)  The method of measuring the outcome is appropriate. | **Low risk:**  No information about the registration protocol was mentioned. But the reported outcomes in the result section seemed to be corresponding with the pre-defined outcomes aforesaid in the method section. | **Some concerns** |
| **Al-Naoum 2014** | **Low risk:**  "Each patient was asked to pick an opaque sealed envelope from a container to allocate the surgical intervention side. The containers included 15 envelopes with the letter ‘R’ indicating the right-hand side and 15 envelopes with the letter ‘L’.” (Page 4) | **Some concerns:**  Blinding cannot be performed.  There is ‘No information’ on whether any deviations arose because of the trial context. | **Some concerns:**  Blinding cannot be performed.  And ‘No information’ on whether the important non-protocol interventions were balanced across intervention groups | **Low risk:**  No dropouts were reported.  “Figure. 1 flow diagram of patients’ recruitment, allocation and follow-up” (Page 21) | **Some concerns:**  No details of blinding of outcome assessors were reported.  The method of measuring the outcome is appropriate. | **Low risk:**  No information about the registration protocol was mentioned. But the reported outcomes in the result section seemed to be corresponding with the pre-defined outcomes aforesaid in the method section. | **Some concerns** |
| **Bhattacharya 2014** | **Some concerns:**  The method used for randomization was not reported.  "Patients were randomized into control and corticotomy groups” (Page 28) | **Some concerns:**  Blinding cannot be performed.  There is ‘No information’ on whether any deviations arose because of the trial context. | **Some concerns:**  Blinding cannot be performed.  And ‘No information’ on whether the important non-protocol interventions were balanced across intervention groups | **Low risk:**  No dropouts were reported. | **Some concerns:**  No details of blinding of outcome assessors were reported.  The method of measuring the outcome is appropriate. | **Low risk:**  No information about the registration protocol was mentioned. But the reported outcomes in the result section seemed to be corresponding with the pre-defined outcomes aforesaid in the method section. | **High risk** |
| **Leethanakul 2014** | **Some concerns:**  The method used for randomization was not reported.  " This study was a split-mouth design in which the experimental side was allocated by randomization” (Page 840) | **Some concerns:**  Blinding cannot be performed.  There is ‘No information’ on whether any deviations arose because of the trial context. | **Some concerns:**  Blinding cannot be performed.  And ‘No information’ on whether the important non-protocol interventions were balanced across intervention groups | **Low risk:**  No dropouts were reported. | **Some concerns:**  No details of blinding of outcome assessors were reported.  The method of measuring the outcome is appropriate. | **Low risk:**  No information about the registration protocol was mentioned. But the reported outcomes in the result section seemed to be corresponding with the pre-defined outcomes aforesaid in the method section. | **High risk** |
| **Al-Naoum 2015** | **Low risk:**  "Each patient was asked to pick an opaque sealed envelope from a container to allocate the treatment approach.” (Page 11) | **Some concerns:**  Blinding cannot be performed.  There is ‘No information’ on whether any deviations arose because of the trial context. | **Some concerns:**  Blinding cannot be performed.  And ‘No information’ on whether the important non-protocol interventions were balanced across intervention groups | **Low risk:**  No dropouts were reported.  “Fig. 1 Participant flow diagram” (Page 11) | **Some concerns:**  No details of blinding of outcome assessors were reported.  The method of measuring the outcome is appropriate. | **Low risk:**  No information about the registration protocol was mentioned. But the reported outcomes in the result section seemed to be corresponding with the pre-defined outcomes aforesaid in the method section. | **Some concerns** |
| **Sharma 2015** | **Some concerns:**  The method used for randomization was not reported.  "Patients were grouped randomly in 3 groups (Page 139) | **Some concerns:**  Blinding cannot be performed.  There is ‘No information’ on whether any deviations arose because of the trial context. | **Some concerns:**  Blinding cannot be performed.  And ‘No information’ on whether the important non-protocol interventions were balanced across intervention groups | **Low risk:**  No dropouts were reported. | **Some concerns:**  No details of blinding of outcome assessors were reported.  The method of measuring the outcome is appropriate. | **Low risk:**  No information about the registration protocol was mentioned. But the reported outcomes in the result section seemed to be corresponding with the pre-defined outcomes aforesaid in the method section. | **High risk** |
| **Suryavanshi 2015** | **Some concerns:**  The method used for randomization was not reported.  " A random sampling method was followed for sampling.” (Page 139) | **Some concerns:**  Blinding cannot be performed.  There is ‘No information’ on whether any deviations arose because of the trial context. | **Some concerns:**  Blinding cannot be performed.  And ‘No information’ on whether the important non-protocol interventions were balanced across intervention groups | **Low risk:**  No dropouts were reported. | **High risk:**  No details of blinding of outcome assessors were reported.  Insufficient information about the method of measuring the outcome. | **Low risk:**  No information about the registration protocol was mentioned. But the reported outcomes in the result section seemed to be corresponding with the pre-defined outcomes aforesaid in the method section. | **High risk** |
| **Abbas 2016** | **Low risk:**  "The randomization was performed with coin tosses to prevent selection bias.” (Page 474) | **Some concerns:**  Blinding cannot be performed.  There is ‘No information’ on whether any deviations arose because of the trial context. | **Some concerns:**  Blinding cannot be performed.  And ‘No information’ on whether the important non-protocol interventions were balanced across intervention groups | **Low risk:**  No dropouts were reported. | **Some concerns:**  No details of blinding of outcome assessors were reported.  The method of measuring the outcome is appropriate. | **Low risk:**  No information about the registration protocol was mentioned. But the reported outcomes in the result section seemed to be corresponding with the pre-defined outcomes aforesaid in the method section. | **Some concerns** |
| **Aksakalli 2016** | **Some concerns:**  The method used for randomization was not reported.  "The study involved a split-mouth design, with the experimental quadrant selected by randomization.” (Page 2) | **Some concerns:**  Blinding cannot be performed.  There is ‘No information’ on whether any deviations arose because of the trial context. | **Some concerns:**  Blinding cannot be performed.  And ‘No information’ on whether the important non-protocol interventions were balanced across intervention groups | **Low risk:**  No dropouts were reported. | **Low risk:**  "The examiner who was responsible for the measurements was blinded.” (Page 3)  The method of measuring the outcome is appropriate. | **Low risk:**  No information about the registration protocol was mentioned. But the reported outcomes in the result section seemed to be corresponding with the pre-defined outcomes aforesaid in the method section. | **Some concerns** |
| **Al-Ainawi 2016** | **Low risk:**  "Each patient was asked to pick an opaque, sealed envelope from a container to allocate the modified distractor side. The container included five envelopes with the letter ‘R’, indicating the right side and five envelopes with the letter ‘L’ for the left side.” (Page 50) | **Some concerns:**  Blinding cannot be performed.  There is ‘No information’ on whether any deviations arose because of the trial context. | **Some concerns:**  Blinding cannot be performed.  And ‘No information’ on whether the important non-protocol interventions were balanced across intervention groups | **Some concerns:**  “three patients were lost to follow-up.” (Page 50) | **Some concerns:**  “Digitization, tracing and analysis of the blinded radiographs were performed by the first author” (Page 53)  No details of blinding of outcome assessors for the other studied variables were reported.  The method of measuring the outcome is appropriate. | **Low risk:**  The protocol for the study was registered on clinical trial.gov with study ID: (NCT02332421) and the outcomes mentioned in the protocol have been reported (Except for patient acceptance to each distractor). | **High risk** |
| **Al-Magaleh 2016** | **Low risk:**  " The subjects were randomly assigned to the split mouth intervention using Excel software program (Microsoft, Inc, Redmond, WA, USA) by a neutral staff member. The randomization lists were concealed in closed envelops and sent to the surgeon.” (Page 3) | **Some concerns:**  Blinding cannot be performed.  There is ‘No information’ on whether any deviations arose because of the trial context. | **Some concerns:**  Blinding cannot be performed.  And ‘No information’ on whether the important non-protocol interventions were balanced across intervention groups | **Low risk:**  No dropouts were reported. | **Some concerns:**  No details of blinding of outcome assessors were reported.  The method of measuring the outcome is appropriate. | **Low risk:**  No information about the registration protocol was mentioned. But it is a preliminary study that does not necessarily need to be registered. | **Some concerns** |
| **Aristizabal 2016** | **Some concerns:**  The method used for randomization was not reported.  "Patients were randomly divided into two groups.” (Page 120) | **Some concerns:**  Blinding cannot be performed.  There is ‘No information’ on whether any deviations arose because of the trial context. | **Some concerns:**  Blinding cannot be performed.  And ‘No information’ on whether the important non-protocol interventions were balanced across intervention groups | **Low risk:**  No dropouts were reported.  “All patients completed the trial and received follow-up care” (Page 122) | **Some concerns:**  No details of blinding of outcome assessors were reported.  The method of measuring the outcome is appropriate. | **Low risk:**  No information about the registration protocol was mentioned. But it is a preliminary study that does not necessarily need to be registered. | **High risk** |
| **Bahammam 2016** | **Low risk:**  "Randomisation was performed using a commercially available computer software package (NCSS-PASS, Number Cruncher Statistical Systems, Kaysville, UT, USA).” (Page 3) | **Some concerns:**  Blinding cannot be performed.  There is ‘No information’ on whether any deviations arose because of the trial context. | **Some concerns:**  Blinding cannot be performed.  And ‘No information’ on whether the important non-protocol interventions were balanced across intervention groups | **Low risk:**  No dropouts were reported. | **Low risk:**  "Another calibrated operator, who was not involved in the study, performed all the clinical and radiographic measurements without knowledge of group assignment.” (Page 4)  The method of measuring the outcome is appropriate. | **Low risk:**  The protocol for the study was registered in clinical trial.gov with study ID: (NCT02796911). | **Some concerns** |
| **Charavet 2016** | **Some concerns:**  The method used for randomization was not reported.  “This study was designed as a randomized controlled clinical trial” (Page 2) | **Some concerns:**  Blinding cannot be performed.  There is ‘No information’ on whether any deviations arose because of the trial context. | **Some concerns:**  Blinding cannot be performed.  And ‘No information’ on whether the important non-protocol interventions were balanced across intervention groups | **Low risk:**  No dropouts were reported. | **Some concerns:**  No details of blinding of outcome assessors were reported.  The method of measuring the outcome is appropriate. | **Low risk:**  No information about the registration protocol was mentioned. But the reported outcomes in the result section seemed to be corresponding with the pre-defined outcomes aforesaid in the method section. | **High risk** |
| **El-Kilani 2016** | **Low risk:**  " The participants were randomly assigned to the test or control groups by Microsoft Excel 2010.” (Page 78) | **Some concerns:**  Blinding cannot be performed.  There is ‘No information’ on whether any deviations arose because of the trial context. | **Some concerns:**  Blinding cannot be performed.  And ‘No information’ on whether the important non-protocol interventions were balanced across intervention groups | **Low risk:**  No dropouts were reported. | **Some concerns:**  No details of blinding of outcome assessors were reported.  The method of measuring the outcome is appropriate. | **Low risk:**  No information about the registration protocol was mentioned. But the reported outcomes in the result section seemed to be corresponding with the pre-defined outcomes aforesaid in the method section. | **Some concerns** |
| **Ersahan 2016** | **Some concerns:**  The method used for randomization was not reported.  “Study participants were randomly divided into a study group of patients” (Page 790) | **Some concerns:**  Blinding cannot be performed.  There is ‘No information’ on whether any deviations arose because of the trial context. | **Some concerns:**  Blinding cannot be performed.  And ‘No information’ on whether the important non-protocol interventions were balanced across intervention groups | **Low risk:**  No dropouts were reported. | **Some concerns:**  No details of blinding of outcome assessors were reported.  The method of measuring the outcome is appropriate. | **Low risk:**  No information about the registration protocol was mentioned. But the reported outcomes in the result section seemed to be corresponding with the pre-defined outcomes aforesaid in the method section. | **High risk** |
| **Jahanbakhshi 2016** | **Some concerns:**  The method used for randomization was not reported.  “one maxillary quadrant was randomly assigned to have corticotomy procedure” (Page 305) | **Some concerns:**  Blinding cannot be performed.  There is ‘No information’ on whether any deviations arose because of the trial context. | **Some concerns:**  Blinding cannot be performed.  And ‘No information’ on whether the important non-protocol interventions were balanced across intervention groups | **Low risk:**  No dropouts were reported. | **High risk:**  No details of blinding of outcome assessors were reported.  Insufficient information about the method of measuring the outcome. | **Low risk:**  The protocol for the study was registered in the Iranian Registry of Clinical Trials with study ID:  (IRCT2013082014415N1). | **High risk** |
| **Eid 2017** | **Low risk:**  "Micro-osteoperforations were randomly assigned (Computer-aided randomization) to the patients’ right or left sides.” (Page 57) | **Some concerns:**  Blinding cannot be performed.  There is ‘No information’ on whether any deviations arose because of the trial context. | **Some concerns:**  Blinding cannot be performed.  And ‘No information’ on whether the important non-protocol interventions were balanced across intervention groups | **Low risk:**  No dropouts were reported. | **Some concerns:**  No details of blinding of outcome assessors were reported.  The method of measuring the outcome is appropriate. | **Low risk:**  No information about the registration protocol was mentioned. But the reported outcomes in the result section seemed to be corresponding with the pre-defined outcomes aforesaid in the method section. | **Some concerns** |
| **Narendra 2017** | **Some concerns:**  The method used for randomization was not reported.  “Group A1 included randomly selected patients for the procedure of periodontally accelerated osteogenic orthodontics. Group A2 included randomly selected patients for the procedure of surgical periodontics for accelerated orthodontics” (Page 2872) | **Some concerns:**  Blinding cannot be performed.  There is ‘No information’ on whether any deviations arose because of the trial context. | **Some concerns:**  Blinding cannot be performed.  And ‘No information’ on whether the important non-protocol interventions were balanced across intervention groups | **Low risk:**  No dropouts were reported. | **Some concerns:**  No details of blinding of outcome assessors were reported.  The method of measuring the outcome is appropriate. | **Low risk:**  No information about the registration protocol was mentioned. But the reported outcomes in the result section seemed to be corresponding with the pre-defined outcomes aforesaid in the method section. | **High risk** |
| **Tunçer 2017** | **Low risk:**  "Randomization was accomplished using opaque, sealed envelopes.” (Page 4) | **Some concerns:**  “Blinding of either the investigator performing the clinical procedures (N.I.T.) or patients was not possible.” (Page 4)  There is ‘No information’ on whether any deviations arose because of the trial context. | **Some concerns:**  “Blinding of either the investigator performing the clinical procedures (N.I.T.) or patients was not possible.” (Page 4)  And ‘No information’ on whether the important non-protocol interventions were balanced across intervention groups | **Low risk:**  "2 patients (one patient in each group) were lost to follow up due to personal reasons.” (Page 5)  Nearly all” the outcome data is available. | **High risk:**  "data assessment was blinded” (Page 4)  "all measurements were done in the patients’ mouth and for right and left sides separately” (Page 3)  This method has poor validity. | **Low risk:**  "The trial was not registered” (Page 1)  However, the reported outcomes in the result section seemed to be corresponding with the pre-defined outcomes aforesaid in the method section. | **High risk** |
| **Uribe 2017** | **Low risk:**  "Randomization sequences were generated using Random Allocation Software program. Random block sizes of six and eight and allocation ratio of 1:1 were generated to ensure balanced numbers in each group. The allocation sequences were sealed around with aluminium foil in envelopes with identical appearance, and were stored in a box.” (Page 2) | **Some concerns:**  Blinding cannot be performed.  There is ‘No information’ on whether any deviations arose because of the trial context. | **Some concerns:**  Blinding cannot be performed.  And ‘No information’ on whether the important non-protocol interventions were balanced across intervention groups | **High risk:**  "Three subjects were lost to follow up.” (Page 4)  The results can be biased by the missing data.  Allocated patients were 19: 16, but the analyzed patients were  16:13. | **Low risk:**  "Two blinded outcome assessors, different from the study coordinator were calibrated in the assessment of the Little’s irregularity index.” (Page 2)  The method of measuring the outcome is appropriate and the outcome assessor was blind. | **Low risk:**  The protocol for the study was registered in clinical trial.gov with study ID: (NCT02026258). | **High risk** |
| **Abdelhameed and Refai 2018** | **Low risk:**  “Assignment of patients and the sides of interventions were performed as following; Computer-generated random numbers were done using Microsoft Office Excel 2013 sheet.” (Page 2181) | **Some concerns:**  Blinding cannot be performed.  There is ‘No information’ on whether any deviations arose because of the trial context. | **Some concerns:**  Blinding cannot be performed.  And ‘No information’ on whether the important non-protocol interventions were balanced across intervention groups. | **Low risk:**  “During the study, there was one dropout patient in (Group C). Also, there were some missing appointments which were all recorded as follows; Group (A), two missing patient appointments at the 4th, and 10th weeks. Group (B), one missing patient appointment in the 10th week. Group (C), no missing patient appointments but there was one dropout patient as mentioned previously.” (Page 2182)  Nearly all” the outcome data is available. | **High risk:**  "Data for the evaluation of each intervention were collected by direct intra-oral measurements” (Page 2182)  The method of measuring the outcome has poor validity and the outcome assessor wasn’t blind. | **Low risk:**  No information about the registration protocol was mentioned. But the reported outcomes in the result section seemed to be corresponding with the pre-defined outcomes aforesaid in the method section. | **High risk** |
| **Alfawal 2018** | **Low risk:**  "Simple randomization was conducted by one of the academic stuff (not involved in this research) at the Department of Orthodontics using computer-generated random numbers with an allocation ratio of 1:1. Allocation sequence was concealed using sequentially numbered, opaque, sealed envelopes, which were opened only after the completion of leveling and alignment stage of the dental arches.” (Page 3) | **Some concerns:**  “Blinding of personnel and participants were not applicable.” (Page 3)  There is ‘No information’ on whether any deviations arose because of the trial context. | **Some concerns:**  “Blinding of personnel and participants were not applicable.” (Page 3)  And ‘No information’ on whether the important non-protocol interventions were balanced across intervention groups | **Low risk:**  "2 patients (one patient in each group) were lost to follow up due to personal reasons.” (Page 5)  Nearly all” the outcome data is available. | **Low risk:**  "blinding was applied only for outcomes’ assessor” (Page 3)  The method of measuring the outcome is appropriate and the outcome assessor was blind. | **Low risk:**  The protocol for the study was registered on clinical trial.gov with study ID: (NCT02606331) and the outcomes mentioned in the protocol have been reported (Except for a change in the levels of pain and discomfort). | **Some concerns** |
| **Al-Jundi 2018** | **Low risk:**  "Each patient was asked to pick an opaque sealed envelope from a container to allocate the ER:YAG laser group.” (Page 195) | **Some concerns:**  Blinding of participants and practitioners cannot be performed.  There is ‘No information’ on whether any deviations arose because of the trial context. | **Some concerns:**  Blinding of participants and practitioners cannot be performed.  And ‘No information’ on whether the important non-protocol interventions were balanced across intervention groups | **Low risk:**  No dropouts were reported. | **Some concerns:**  No details of blinding of outcome assessors were reported.  The method of measuring the outcome is appropriate. | **Low risk:**  No information about the registration protocol was mentioned. But the reported outcomes in the result section seemed to be corresponding with the pre-defined outcomes aforesaid in the method section. | **Some concerns** |
| **Alkebsi 2018** | **Low risk:**  "The randomization was accomplished by using the permuted random block size of 2 with the random generation function in Excel (Microsoft, Redmond, Wash).” (Page 773) | **Some concerns:**  “Blinding of either patient or clinician was not possible.” (Page 773)  There is ‘No information’ on whether any deviations arose because of the trial context. | **Some concerns:**  “Blinding of either patient or clinician was not possible.” (Page 773)  And ‘No information’ on whether the important non-protocol interventions were balanced across intervention groups | **Low risk:**  "Three subjects were excluded after MOP intervention due to either irregular attendance or poor oral hygiene.” (Page 778)  Nearly all” the outcome data is available. | **Low risk:**  "Blinding was ensured at the measurement stage (data collection)” (Page 773)  The method of measuring the outcome is appropriate and the outcome assessor was blind. | **Low risk:**  The protocol for the study was registered on clinical trial.gov with study ID: (NCT02473471) and the outcomes mentioned in the protocol have been reported (Except for the relationship between the rate of tooth movement and Menstrual cycle). | **Some concerns** |
| **Attri 2018** | **Low risk:**  "Randomisation was carried out, using a computer-generated random allocation sequence (RAND function-Microsoft Excel 2010) to ensure equivalence of numbers in each group. The sequences were concealed in envelopes, which were chosen by the patient. This was carried out independently and the primary clinical investigators had no role in this” (Page 4) | **Some concerns:**  “Blinding of the participating patient and the treating clinician was not possible.” (Page 4)  There is ‘No information’ on whether any deviations arose because of the trial context. | **Some concerns:**  “Blinding of the participating patient and the treating clinician was not possible.” (Page 4)  And ‘No information’ on whether the important non-protocol interventions were balanced across intervention groups | **Low risk:**  "No subjects were lost to follow up.” (Page 4) | **Low risk:**  " Only the data analyzer was blinded for treatment groups and treatment time points)” (Page 4)  The method of measuring the outcome is appropriate and the outcome assessor was blind. | **Low risk:**  The protocol for the study was registered in ctri.nic.in. A wrong ID number of the registration protocol was reported in the article (CTRI/2018/03/XXXXXX).  The correct ID seems to be CTRI/2018/03/012331 and the outcomes mentioned in the protocol have been reported (Except for incisor irregularity). | **Some concerns** |
| **Chandran 2018** | **Some concerns:**  The method used for randomization was not reported.  “The subjects were selected randomly” (Page 129) | **Some concerns:**  Blinding cannot be performed.  There is ‘No information’ on whether any deviations arose because of the trial context. | **Some concerns:**  Blinding cannot be performed.  And ‘No information’ on whether the important non-protocol interventions were balanced across intervention groups. | **Low risk:**  No dropouts were reported. | **Some concerns:**  No details of blinding of outcome assessors were reported.  The method of measuring the outcome is appropriate. | **Low risk:**  No information about the registration protocol was mentioned. But the reported outcomes in the result section seemed to be corresponding with the pre-defined outcomes aforesaid in the method section. | **High risk** |
| **Elkalza 2018** | **Low risk:**  "The patients were randomly allocated by a sequence generated in SPSS and the allocation was centrally concealed.” (Page 24) | **Some concerns:**  Blinding of participants and practitioners cannot be performed.  There is ‘No information’ on whether any deviations arose because of the trial context. | **Some concerns:**  Blinding of participants and practitioners cannot be performed.  And ‘No information’ on whether the important non-protocol interventions were balanced across intervention groups | **Low risk:**  No dropouts were reported. | **Some concerns:**  No details of blinding of outcome assessors were reported.  The method of measuring the outcome is appropriate. | **Low risk:**  No information about the registration protocol was mentioned. But the reported outcomes in the result section seemed to be corresponding with the pre-defined outcomes aforesaid in the method section. | **Some concerns** |
| **Gibreal 2018** | **Low risk:**  "With the aid of computer-generated list of random numbers, the recruited patients were assigned to two parallel groups with a 1:1 allocation ratio. Allocation sequence was concealed using sequentially numbered, opaque, sealed envelopes which were opened only after the completion of premolars extraction.” (Page 2) | **Some concerns:**  “Blinding of personnel and participants were not applicable.” (Page 2)  There is ‘No information’ on whether any deviations arose because of the trial context. | **Some concerns:**  “Blinding of personnel and participants were not applicable.” (Page 2)  And ‘No information’ on whether the important non-protocol interventions were balanced across intervention groups | **Low risk:**  “One female patient was lost to follow-up from the control group, and another female patient was excluded from analysis for the experimental group” (Page 4)  “Nearly all” the outcome data is available. | **Low risk:**  "blinding was applied only for outcomes’ assessor” (Page 2)  The method of measuring the outcome is appropriate and the outcome assessor was blind. | **Low risk:**  The protocol for the study was registered on clinical trial.gov with study ID: (NCT02977221). | **Some concerns** |
| **Haliloglu-Ozkan 2018** | **Some concerns:**  The method used for randomization was not reported.  “This randomized, single-center, single-blinded study” (Page e762) | **Some concerns:**  “A single examiner administering the treatment was aware of the inclusion of subjects into groups and was therefore not blinded.” (Page e762)  There is ‘No information’ on whether any deviations arose because of the trial context. | **Some concerns:**  “A single examiner administering the treatment was aware of the inclusion of subjects into groups and was therefore not blinded.” (Page e762)  And ‘No information’ on whether the important non-protocol interventions were balanced across intervention groups | **Some concerns:**  “One patient in the OP group and three in the control group were  excluded from the study” (Page e762) | **Low risk:**  "A single examiner responsible for the digital measurements and data analysis was blinded.” (Page e763)  The method of measuring the outcome is appropriate and the outcome assessor was blind. | **Low risk:**  No information about the registration protocol was mentioned. But the reported outcomes in the result section seemed to be corresponding with the pre-defined outcomes aforesaid in the method section. | **High risk** |
| **Khalil 2018** | **Low risk:**  "Simple randomization was designed with the aid of computer generated schedule in Excel with 1:1 allocation ratio followed by allocation concealment.” (Page 13) | **Some concerns:**  “Blinding of the operators was not applicable.” (Page 13)  There is ‘No information’ on whether any deviations arose because of the trial context. | **Some concerns:**  “Blinding of the operators was not applicable.” (Page 13)  And ‘No information’ on whether the important non-protocol interventions were balanced across intervention groups | **Low risk:**  No dropouts were reported. | **Low risk:**  "blinding was only limited to the outcome assessment.” (Page 13)  The method of measuring the outcome is appropriate and the outcome assessor was blind. | **Low risk:**  No information about the registration protocol was mentioned. But the reported outcomes in the result section seemed to be corresponding with the pre-defined outcomes aforesaid in the method section. | **Some concerns** |
| **Kundi 2018** | **Low risk:**  "The patients were randomly allocated by a sequence generated in SPSS with equal number of participants in each group, and the allocation was centrally concealed.” (Page 310) | **Some concerns:**  “patient and operator blinding was not possible.” (Page 310)  There is ‘No information’ on whether any deviations arose because of the trial context. | **Some concerns:**  “patient and operator blinding was not possible.” (Page 310)  And ‘No information’ on whether the important non-protocol interventions were balanced across intervention groups | **Low risk:**  "There were no losses to follow up.” (Page 312) | **Low risk:**  "Blinding was carried out at the analysis stage” (Page 310)  The method of measuring the outcome is appropriate and the outcome assessor was blind. | **Low risk:**  No information about the registration protocol was mentioned. But the reported outcomes in the result section seemed to be corresponding with the pre-defined outcomes aforesaid in the method section. | **Some concerns** |
| **Thind 2018** | **Some concerns:**  The method used for randomization was not reported.  “This is a randomized case‑controlled clinical trial performed on 40 participants” (Page 329) | **Some concerns:**  Blinding of participants and practitioners cannot be performed.  There is ‘No information’ on whether any deviations arose because of the trial context. | **Some concerns:**  Blinding of participants and practitioners cannot be performed.  And ‘No information’ on whether the important non-protocol interventions were balanced across intervention groups | **Low risk:**  "A total of 40 participants completed the entire duration of the study.” (Page 329) | **High risk:**  No details of blinding of outcome assessors were reported.  Insufficient information about the method of measuring the outcome. | **Some concerns:**  No information about the registration protocol was mentioned.  Moreover, not all outcome variables to be studied, may not have been reported as the detailes reporting in the Methods section were insufficient. | **High risk** |
| **Aboalnaga 2019** | **Low risk:**  "Computer-generated random numbers were generated using Microsoft Office Excel 2007 sheet by a person who was not involved in the clinical trial (MA). The patients’ right sides were randomly assigned to either the MOP or control groups.” (Page 2) | **Some concerns:**  Blinding of participants and practitioners cannot be performed.  There is ‘No information’ on whether any deviations arose because of the trial context. | **Some concerns:**  Blinding of participants and practitioners cannot be performed.  And ‘No information’ on whether the important non-protocol interventions were balanced across intervention groups | **Low risk:**  "All 18 patients had successfully completed the 4 months duration of the study.” (Page 4) | **Low risk:**  "The primary assessor (N.A) performed digital models and CBCT measurements blindly for each patient” (Page 3)  The method of measuring the outcome is appropriate and the outcome assessor was blind. | **Low risk:**  The protocol for the study was registered on clinical trial.gov with study ID: (NCT03450278). | **Some concerns** |
| **Agrawal 2019** | **Low risk:**  "Corticotomy site and MOP’s site (in total 20 sites) were assigned randomly by envelope method in each patient.” (Page 2) | **Some concerns:**  Blinding of participants and practitioners cannot be performed.  There is ‘No information’ on whether any deviations arose because of the trial context. | **Some concerns:**  Blinding of participants and practitioners cannot be performed.  And ‘No information’ on whether the important non-protocol interventions were balanced across intervention groups | **Low risk:**  No dropouts were reported. | **Some concerns:**  No details of blinding of outcome assessors were reported.  The method of measuring the outcome is appropriate. | **Low risk:**  No information about the registration protocol was mentioned. But the reported outcomes in the result section seemed to be corresponding with the pre-defined outcomes aforesaid in the method section. | **Some concerns** |
| **Al-Imam 2019** | **Low risk:**  "Simple randomization was done by one member of the academic staff not involved in this trial, using a computer generated list of random numbers (Minitab, v. 17). Allocation was concealed using sequentially numbered, opaque, sealed envelopes, which were opened only after the end of the canine retraction phase.” (Page 387) | **Some concerns:**  “The blinding of the principal investigator and the patients was impossible.” (Page 387)  There is ‘No information’ on whether any deviations arose because of the trial context. | **Some concerns:**  “The blinding of the principal investigator and the patients was impossible.” (Page 387)  And ‘No information’ on whether the important non-protocol interventions were balanced across intervention groups | **Low risk:**  “One patient withdrew from CG for personal reasons and another patient was excluded from the experimental group” (Page 390)  “Nearly all” the outcome data is available. | **Low risk:**  "blinding was only employed in the data analysis” (Page 387)  The method of measuring the outcome is appropriate and the outcome assessor was blind. | **Low risk:**  The protocol for the study was registered on clinical trial.gov with study ID: (NCT03149016), and the outcomes mentioned in the protocol have been reported (Except for the tipping, torque, and rotation of maxillary canine and first molar) | **Some concerns** |
| **Alqadasi 2019** | **Low risk:**  "The intervention was randomly allocated to either right or left side with 1:1 allocation ratio. To increase the unpredictability of the random allocation sequence, the sequences with either right or left were concealed in opaque envelope and shuffled.” (Page 639) | **Some concerns:**  Blinding of participants and practitioners cannot be performed.  There is ‘No information’ on whether any deviations arose because of the trial context. | **Some concerns:**  Blinding of participants and practitioners cannot be performed.  And ‘No information’ on whether the important non-protocol interventions were balanced across intervention groups | **Low risk:**  No dropouts were reported. | **Low risk:**  "Blinding was used at the data collection and analysis stages.” (Page 637)  The method of measuring the outcome is appropriate and the outcome assessor was blind. | **Low risk:**  No information about the registration protocol was mentioned. But the reported outcomes in the result section seemed to be corresponding with the pre-defined outcomes aforesaid in the method section. | **Some concerns** |
| **Bansal 2019** | **Low risk:**  "Stratified randomization method was used to ensure a 1:1 allocation ratio and allocation concealment was achieved with similar looking sealed opaque envelopes.” (Page 2) | **Some concerns:**  “The subjects and the operator administering treatment were aware of the group assignment and therefore were not blinded.” (Page 4)  There is ‘No information’ on whether any deviations arose because of the trial context. | **Some concerns:**  “The subjects and the operator administering treatment were aware of the group assignment and therefore were not blinded.” (Page 4)  And ‘No information’ on whether the important non-protocol interventions were balanced across intervention groups | **Low risk:**  “No dropout occurred, and complete follow‑up and analysis were achieved for all patients” (Page 4) | **Low risk:**  "the investigators performing the measurements and data analysis were blinded from the group assignments” (Page 4)  The method of measuring the outcome is appropriate and the outcome assessor was blind. | **Low risk:**  The protocol for the study was registered in International Clinical Trials Registry Platform Search Portal with study ID: (CTRI/2017/11/010610). | **Some concerns** |
| **Chandra 2019** | **Low risk:**  "Using block randomization, individuals were randomly divided into two groups.” (Page 2) | **Low risk:**  “The orthodontist who performed the relevant treatment after PAOO was different from the orthodontist randomizing the individuals into C or C + BMP groups and hence was blinded to the treatment received by the patient.” (Page 2) | **Low risk:**  “The orthodontist who performed the relevant treatment after PAOO was different from the orthodontist randomizing the individuals into C or C + BMP groups and hence was blinded to the treatment received by the patient.” (Page 2) | **Low risk:**  “there were no dropouts” (Page 4) | **Low risk:**  "another periodontist blinded to the therapy received by the individuals recorded the clinical and radiographic data pertinent to the study.” (Page 2)  The method of measuring the outcome is appropriate and the outcome assessor was blind. | **Low risk:**  No information about the registration protocol was mentioned. But the reported outcomes in the result section seemed to be corresponding with the pre-defined outcomes aforesaid in the method section. | **Low risk** |
| **Charavet 2019**  **(PROMs)** | **Low risk:**  "Sealed envelopes containing the random allocation of each patient to one or the other group were prepared by an independent team and opened as patients accrued.” (Page 3) | **Some concerns:**  “the patients were not blinded regarding their groups. Furthermore, the orthodontic operator could not be entirely blinded since it was impossible to hide the piezocision scars when visible.” (Page 7)  There is ‘No information’ on whether any deviations arose because of the trial context. | **Some concerns:**  “the patients were not blinded regarding their groups. Furthermore, the orthodontic operator could not be entirely blinded since it was impossible to hide the piezocision scars when visible.” (Page 7)  And ‘No information’ on whether the important non-protocol interventions were balanced across intervention groups | **Low risk:**  No dropouts were reported.  “Fig. 1 Prisma flow diagram” (Page 2) | **High risk:**  No details of blinding of outcome assessors were reported.  There is a difference in the assessment time points (the control group was assessed for 7 days after appliance bonding, while the intervention group was assessed 7 days after bonding and also after applying the surgical intervention. (Page 4) | **Low risk:**  The protocol for the study was registered on clinical trial.gov with study ID: (NCT03406130). | **High risk** |
| **Charavet 2019** | **Low risk:**  "a sealed envelope with the corresponding number was opened to disclose the allocated group (control or test).” (Page 3) | **Some concerns:**  Blinding cannot be performed.  There is ‘No information’ on whether any deviations arose because of the trial context. | **Some concerns:**  Blinding cannot be performed.  And ‘No information’ on whether the important non-protocol interventions were balanced across intervention groups. | **Low risk:**  “Two patients (one in each group) failed to attend the post-treatment CBCT scan.” (Page 4)  “Nearly all” the outcome data is available. | **Some concerns:**  No details of blinding of outcome assessors were reported.  The method of measuring the outcome is appropriate. | **Low risk:**  The protocol for the study was registered on clinical trial.gov with study ID: (NCT03406130). | **Some concerns** |
| **Chourasia 2019** | **Low risk:**  "Randomization was performed using a software-generated list of random numbers; the recruited patients were divided into two parallel groups with a 1:1 allocation ratio.” (Page 7) | **Some concerns:**  Blinding of participants and practitioners cannot be performed.  There is ‘No information’ on whether any deviations arose because of the trial context. | **Some concerns:**  Blinding of participants and practitioners cannot be performed.  And ‘No information’ on whether the important non-protocol interventions were balanced across intervention groups | **Low risk:**  No dropouts were reported. | **High risk:**  "Assessor blinding was employed.” (Page 7)  Insufficient information about the method of measuring the outcome. | **Some concerns:**  No information about the registration protocol was mentioned.  Moreover, not all outcome variables to be studied, may not have been reported, as the details reported in the Methods section were insufficient. | **High risk** |
| **Deepak 2019** | **Low risk:**  "the group was selected by picking of lot.” (Page 2873) | **Some concerns:**  Blinding of participants and practitioners cannot be performed.  There is ‘No information’ on whether any deviations arose because of the trial context. | **Some concerns:**  Blinding of participants and practitioners cannot be performed.  And ‘No information’ on whether the important non-protocol interventions were balanced across intervention groups | **Low risk:**  “1 patient discontinued the study due to personal reasons.” (Page 2873)  “Nearly all” the outcome data is available. | **High risk:**  No details of blinding of outcome assessors were reported.  Insufficient information about the method of measuring the outcome. | **Some concerns:**  No information about the registration protocol was mentioned.  Moreover, not all outcome variables to be studied, may not have been reported, as the details reported in the Methods section were insufficient. | **High risk** |
| **Gibreal 2019** | **Low risk:**  "Patients were assigned to the experimental group or the control group with an allocation ratio of 1:1 using a software-generated list of random numbers. Allocation sequence was concealed using sequentially numbered, opaque, sealed envelopes which were opened only after the completion of premolars extraction.” (Page 4) | **Some concerns:**  “blinding was neither applied to the researcher nor to the patients during this trial.” (Page 8)  There is ‘No information’ on whether any deviations arose because of the trial context. | **Some concerns:**  “blinding was neither applied to the researcher nor to the patients during this trial.” (Page 8)  And ‘No information’ on whether the important non-protocol interventions were balanced across intervention groups | **Low risk:**  “one patient in each group dropped out before the end of the trial due to personal reasons” (Page 5)  “Nearly all” the outcome data is available. | **Low risk:**  "Single blinding was employed in this trial regarding outcome measure assessment and data analysis.” (Page 4)  The method of measuring the outcome is appropriate and the outcome assessor was blind. | **Low risk:**  The protocol for the study was registered in clinical trial.gov with study ID: (NCT02975765). | **Some concerns** |
| **Kumar 2019** | **Low risk:**  " Adequate method was used to generate the random allocation  sequence and block randomization was selected. The method to be used for random sequence was coin toss.” (Page 2) | **Some concerns:**  Blinding of participants and practitioners cannot be performed.  There is ‘No information’ on whether any deviations arose because of the trial context. | **Some concerns:**  Blinding of participants and practitioners cannot be performed.  And ‘No information’ on whether the important non-protocol interventions were balanced across intervention groups | **Low risk:**  No dropouts were reported.  “Figure 1: Flowchart showing the study layout” (Page 2) | **High risk:**  No details of blinding of outcome assessors were reported.  Insufficient information about the method of measuring the rate of canine retraction. | **Low risk:**  The protocol for the study was registered at WHO – Clinical Trials Registry – India with study ID: (CTRI/2017/05/008496). | **High risk** |
| **Rathod 2019** | **Some concerns:**  The method used for randomization was not reported.  “Each patient’s maxillary arch was divided into the right and left quadrants. Experimental and conventional sides were randomly allocated” (Page 91) | **Some concerns:**  Blinding of participants and practitioners cannot be performed.  There is ‘No information’ on whether any deviations arose because of the trial context. | **Some concerns:**  Blinding of participants and practitioners cannot be performed.  And ‘No information’ on whether the important non-protocol interventions were balanced across intervention groups | **Low risk:**  No dropouts were reported. | **Some concerns:**  No details of blinding of outcome assessors were reported.  The method of measuring the outcome is appropriate. | **Low risk:**  No information about the registration protocol was mentioned. But the reported outcomes in the result section seemed to be corresponding with the pre-defined outcomes aforesaid in the method section. | **High risk** |
| **Singh 2019** | **Some concerns:**  The method used for randomization was not reported.  “the patients were randomly distributed into two groups” (Page 572) | **Some concerns:**  Blinding of participants and practitioners cannot be performed.  There is ‘No information’ on whether any deviations arose because of the trial context. | **Some concerns:**  Blinding of participants and practitioners cannot be performed.  And ‘No information’ on whether the important non-protocol interventions were balanced across intervention groups | **Low risk:**  No dropouts were reported. | **Some concerns:**  No details of blinding of outcome assessors were reported.  The method of measuring the outcome is appropriate. | **Low risk:**  No information about the registration protocol was mentioned. But the reported outcomes in the result section seemed to be corresponding with the pre-defined outcomes aforesaid in the method section. | **High risk** |
| **Sivarajan 2019** | **Low risk:**  “Randomized block sampling was carried out using RANDOM.ORG online software to allocate participants into three intervention groups on a 1:1:1 basis.” (Page 185) | **Some concerns:**  Blinding cannot be performed.  There is ‘No information’ on whether any deviations arose because of the trial context. | **Some concerns:**  Blinding cannot be performed.  And ‘No information’ on whether the important non-protocol interventions were balanced across intervention groups. | **Low risk:**  “Thirty subjects were enrolled into the study between September 2014 and  March 2016 with data collection complete by March 2017 and no dropouts.” (Page 186)  “All” the outcome data is available. | **High risk:**  “the distance from the central point of the canine bracket to the superior margin of the miniimplant (maxilla) and the inferior margin of the miniimplant (mandible) and the distance from the canine cusp tip to the mesiobuccal groove of the first molar was clinically measured using electric digital calipers (accurate to 0.01 mm).” (Page 185)  This method has poor validity.  “The outcome measurements was also blinded.” (Page 185)  The outcome assessor was blind. | **Some concerns:**  The trial mentioned that the protocol was registered at clinicaltrial.gov by the ID:(DFCD1412/0089P). The ID was not identified at clinical trial.gov. So, the registration protocol could not be retrieved to detect the data that existed. | **High risk** |
| **Yousif 2019** | **Some concerns:**  The method used for randomization was not reported.  “A randomized split mouth clinical multi-operator study was performed  on 30 orthodontic patients” (Page 3223) “Subjects were randomized equally into three canine retraction groups” | **Some concerns:**  Blinding cannot be performed.  There is ‘No information’ on whether any deviations arose because of the trial context. | **Some concerns:**  Blinding cannot be performed.  And ‘No information’ on whether the important non-protocol interventions were balanced across intervention groups. | **Low risk:**  No dropouts were reported. | **High risk:**  “The distance between the distal surface of the canine and the mesial surface of the second premolar was recorded directly in patient’s mouth every week using a caliper with 0.01 mm scale” (Page 3226)  The method of measuring the outcome has poor validity and the outcome assessor wasn’t blind. | **Low risk:**  No information about the registration protocol was mentioned. But the reported outcomes in the result section seemed to be corresponding with the pre-defined outcomes aforesaid in the method section. | **High risk** |
| **Abdarazik 2020** | **Some concerns:**  The method used for randomization was not reported.  “Randomized selection was done for patients that were treated with FTMPF (Full thickness mucopreiosteal flap) and other with LLLT (Low level laser thereby).” (Page 287) | **Some concerns:**  Blinding cannot be performed.  There is ‘No information’ on whether any deviations arose because of the trial context. | **Some concerns:**  Blinding cannot be performed.  And ‘No information’ on whether the important non-protocol interventions were balanced across intervention groups. | **Low risk:**  No dropouts were reported. | **Low risk:**  “For all patients the rate of canine retraction was measured by measuring the distance moved by the maxillary canine through a series of 3D scanned study models and 3D superimposition.” (Page 288) The method of measuring the outcome is appropriate. | **Low risk:**  No information about the registration protocol was mentioned. But the reported outcomes in the result section seemed to be corresponding with the pre-defined outcomes aforesaid in the method section. | **Some concerns** |
| **Alfawal 2020** | **Low risk:**  "Computer-generated randomization lists with an allocation ratio 1:1 were created by one of the co-authors (O.H.) using Minitab®, v. 17 (Minitab Inc., State College, USA). Allocation was concealed by using a sequentially numbered, opaque, sealed envelope.” (Page 2) | **Some concerns:**  “blinding of the principal researcher and the patients was not possible.” (Page 3)  There is ‘No information’ on whether any deviations arose because of the trial context. | **Some concerns:**  “blinding of the principal researcher and the patients was not possible.” (Page 2)  And ‘No information’ on whether the important non-protocol interventions were balanced across intervention groups | **Low risk:**  "All patients completed their questionnaires and no patient was lost to follow-up.” (Page 4) | **Low risk:**  "Blinding was applied only for the outcome assessor” (Page 2)  The method of measuring the outcome is appropriate and the outcome assessor was blind. | **Low risk:**  No information about the registration protocol was mentioned. But the reported outcomes in the result section seemed to be corresponding with the pre-defined outcomes aforesaid in the method section. | **Some concerns** |
| **Asif 2020** | **Some concerns:**  The method used for randomization was not reported.  “This study was a single-center, single-blind, prospective randomized split-mouth clinical trial.” (Page 580) | **Some concerns:**  Blinding cannot be performed.  There is ‘No information’ on whether any deviations arose because of the trial context. | **Some concerns:**  Blinding cannot be performed.  And ‘No information’ on whether the important non-protocol interventions were balanced across intervention groups. | **Some concerns:**  6 patients (2 from MOP 4-weeks Group and 4 from MOP 8-weeks Group) were dropout.  The reasons are illustrated in Figure 4 (Consort flow diagram). (Page 583) | **High risk:**  “The distance of canine movement was recorded every 4 weeks with digital calipers accurate to 0.01 mm, for 12 weeks” (Page 581)  This method has poor validity.  “Two observers (orthodontic postgraduate students) were blinded to the frequency of MOP while analyzing the BV/TV ratio using CT Analyzer software as CBCT files were labeled by random numbers.” (Page 581) | **Low risk:**  No information about the registration protocol was mentioned. But the reported outcomes in the result section seemed to be corresponding with the pre-defined outcomes aforesaid in the method section. | **High risk** |
| **Babanouri 2020** | **Low risk:**  "The allocation of the participants was based on the block randomization method (block length = 4) using the online RANDOM.ORG software. Each random number was placed in a sealed opaque envelope and subsequently. For each participant, by tossing a coin, the MOPs intervention was randomly assigned to the right or left side at first premolar extraction sites, while the opposite side served as the split-mouth control.” (Page 3) | **Low risk:**  “both the patients and the orthodontist were blinded to the experimental side.” (Page 3) | **Low risk:**  “both the patients and the orthodontist were blinded to the experimental side.” (Page 3) | **Low risk:**  “two patients in the MOP1 group and one patient in the MOP2 group were excluded from the study” (Page 5)  “Nearly all” the outcome data is available. | **Low risk:**  "the second author (SHA), responsible for the measurements, and the statistician were blinded to the coding of the study models” (Page 3)  The method of measuring the outcome is appropriate and the outcome assessor was blind. | **Low risk:**  The protocol for the study was registered in the Iranian Registry of Clinical Trials with study ID: (IRCT20181121041713N1). | **Low risk** |
| **El Mahlawy 2020** | **Some concerns:**  The method used for randomization was not reported.  “Randomly the sample was allocated into 2 groups.” (Page 622) | **Some concerns:**  Blinding cannot be performed.  There is ‘No information’ on whether any deviations arose because of the trial context. | **Some concerns:**  Blinding cannot be performed.  And ‘No information’ on whether the important non-protocol interventions were balanced across intervention groups. | **Low risk:**  No dropouts were reported**.** | **Some concerns:**  No details of blinding of outcome assessors were reported.  The method of measuring the outcome is appropriate. | **Low risk:**  No information about the registration protocol was mentioned. But the reported outcomes in the result section seemed to be corresponding with the pre-defined outcomes aforesaid in the method section. | **High risk** |
| **Fattori 2020** | **Some concerns:**  "Randomization was performed with number sequences generated using the Random function in Excel with a 1:1 allocation ratio.” (Page 642)  Although method used for randomization was reported, there baseline differences between intervention and control groups suggest a problem with the allocation sequence process as 13 patients were allocated to control group, while 11 patients were allocated to intervention group. | **Some concerns:**  “Blinding was not possible because of the type of intervention.” (Page 642)  There is ‘No information’ on whether any deviations arose because of the trial context. | **Some concerns:**  “Blinding was not possible because of the type of intervention.” (Page 642)  And ‘No information’ on whether the important non-protocol interventions were balanced across intervention groups | **Some concerns:**  "For the primary outcome, 18 participants were allocated and completed the study, providing 70% of power to detect 0.4 mm of difference.” (Page 642) | **Low risk:**  "To ensure blinding feasibility, data collectors and outcome assessors were blinded for analysis.” (Page 642)  The method of measuring the outcome is appropriate and the outcome assessor was blind. | **Low risk:**  The protocol for the study was registered on clinical trial.gov with study ID: (NCT02416297). | **High risk** |
| **Gulduren 2020** | **Low risk:**  "Randomization was performed using identical, opaque, sealed envelopes [16]. A total of 20 envelopes contained standard-sized treatment allocation papers: 10 allocation papers were for the experimental group (5 for right MOPs, 5 for left MOPs) and 10 allocation papers were for the control group.” (Page 131) | **Some concerns:**  Blinding cannot be performed.  There is ‘No information’ on whether any deviations arose because of the trial context. | **Some concerns:**  Blinding cannot be performed.  And ‘No information’ on whether the important non-protocol interventions were balanced across intervention groups. | **Low risk:**  “One subject from the control group and one subject from the experimental group were excluded from the study due to missing follow-up data.” (Page 132)  “Nearly all” the outcome data is available. | **Low risk:**  "All digital models were coded to ensure the blindness of the investigators while assessing the rate of tooth movement. By coding all the models, the investigator was blinded of the control, experimental groups and the side to which MOPs were applied. The periodontist who performed the periodontal assessments was blinded of the subject groups and applied procedures.” (Page 131)  The method of measuring the outcome is appropriate and the outcome assessor was blind. | **Low risk:**  No information about the registration protocol was mentioned. But the reported outcomes in the result section seemed to be corresponding with the pre-defined outcomes aforesaid in the method section. Moreover, it was reported in the article: “No changes were applied to the methods after trial commencement.” (Page 128) and “No changes were applied to outcomes after trial commencement.” (Page 131) | **Some concerns** |
| **Hatrom 2020** | **Low risk:**  "Patients were randomly assigned to either the PCG or CG using the opaque sealed-envelope technique. Envelopes containing treatment allocation cards were prepared, and the deck of cards was shuffled  thoroughly. After shuffling, patients were asked to pick one of the opaque sealed envelopes from a container to allocate the treatment approach.” (Page 649) | **Some concerns:**  Blinding cannot be performed.  There is ‘No information’ on whether any deviations arose because of the trial context. | **Some concerns:**  Blinding cannot be performed.  And ‘No information’ on whether the important non-protocol interventions were balanced across intervention groups. | **Some concerns:**  "1 patient in the PCG and 2 patients in the CG were excluded after enrollment as a result of miniscrew failure." (Page 651) | **Low risk:**  "Cone-beam computed tomography (CBCT) assessment was blinded” (Page 649)  The method of measuring the outcome is appropriate and the outcome assessor was blind. | **Low risk:**  The protocol for the study was registered on clinical trial.gov with study ID: (NCT03180151). | **Some concerns** |
| **Kandil 2020** | **Low risk:**  "A strict randomization method was used in blocks of 5 to ensure that balanced number of patients was allocated to each treatment group. The principal investigator was blinded to the allocation sequence, which was generated by the statistician in this study using computergenerated random numbers.” (Page 3) | **Some concerns:**  “Blinding was not possible for the operators and the subjects.” (Page 6)  There is ‘No information’ on whether any deviations arose because of the trial context. | **Some concerns:**  “Blinding was not possible for the operators and the subjects.” (Page 6)  And ‘No information’ on whether the important non-protocol interventions were balanced across intervention groups | **Low risk:**  “None of the patients in both treatment groups discontinued the treatment procedure.” (Page 7) | **Low risk:**  "The two assessors blindly and independently, through the random codes given to subjects and numbers given to the models; took the measurements on dental models.” (Page 6)  The method of measuring the outcome is appropriate and the outcome assessor was blind. | **Low risk:**  No information about the registration protocol was mentioned. But the reported outcomes in the result section seemed to be corresponding with the pre-defined outcomes aforesaid in the method section. | **Some concerns** |
| **Khlef 2020** | **Low risk:**  "Simple randomization was conducted by one of the academic staff (not involved in the study) using computer-generated random numbers with an allocation ratio of 1:1. The allocation sequence was concealed using sequentially numbered, opaque, sealed envelopes.” (Page e5) | **Some concerns:**  “Blinding either the investigator performing the clinical procedures (H.N.K) or patients was not possible.” (Page e6)  There is ‘No information’ on whether any deviations arose because of the trial context. | **Some concerns:**  “Blinding either the investigator performing the clinical procedures (H.N.K) or patients was not possible.” (Page e6)  And ‘No information’ on whether the important non-protocol interventions were balanced across intervention groups | **Low risk:**  “no patient was lost to follow-up in both groups.” (Page e6) | **Low risk:**  "blinding was applied only for the outcomes' assessor.” (Page e6)  The method of measuring the outcome is appropriate and the outcome assessor was blind. | **Low risk:**  The protocol for the study was registered on clinical trial.gov with study ID: (NCT03279042). | **Some concerns** |
| **Mahmoudzadeh 2020** | **Low risk:**  "In this study, we considered two treatment blocks for patients. In the first block, the right quadrant was considered as the control side while the left quadrant was considered as the laser side. In the second block, the left quadrant served as the control side and the right quadrant was considered as the laser side. The allocation of patients to the treatment blocks was performed by flipping a coin, which was done by someone not involved in the study.” (Page 444) | **Some concerns:**  “blinding the patients, clinicians, and the research director was not possible due to its specific design.” (Page 445)  There is ‘No information’ on whether any deviations arose because of the trial context. | **Some concerns:**  “blinding the patients, clinicians, and the research director was not possible due to its specific design.” (Page 445)  And ‘No information’ on whether the important non-protocol interventions were balanced across intervention groups. | **Low risk:**  No dropouts were reported**.** | **Low risk:**  "the assessor who analyzed the landmarks and measured the distances was blinded to the group allocation of the samples. Data were statistically analyzed by a statistician who was also blinded to the group allocation of the samples” (Page 445)  The method of measuring the outcome is appropriate and the outcome assessor was blind. | **Low risk:**  The protocol for the study was registered in the Iranian Registry of Clinical Trials available at www.irct.ir (identifier: IRCT20120215009014N280). | **Some concerns** |
| **Mittal 2020** | **Low risk:**  "Block randomization was done to achieve an equal number of participants in both groups. Patient case record numbers were used as input in the allocation sequence. The concealed sequences in sealed envelopes were then chosen by the patient.” (Page 636) | **Some concerns:**  “Blinding of the participants and the primary investigator was not possible.” (Page 637)  There is ‘No information’ on whether any deviations arose because of the trial context. | **Some concerns:**  “Blinding of the participants and the primary investigator was not possible.” (Page 637)  And ‘No information’ on whether the important non-protocol interventions were balanced across intervention groups. | **Low risk:**  “none of the participants were lost during the trial.” (Page 637) | **Low risk:**  "The models were coded to blind the investigator performing data analysis to the identity of the two groups.” (Page 637)  The method of measuring the outcome is appropriate and the outcome assessor was blind. | **Some concerns:**  The protocol for the study was registered at the National Trial Registry with study ID: (CTRI/2018/03/012331), and the outcomes mentioned in the protocol have been reported (Except for the alignment efficiency,irregularity index, and pain perception). | **Some concerns** |
| **Omidkhoda 2020** | **Low risk:**  “A random selection procedure (coin toss) was utilized to consider one side of the participant’s upper jaw for piezo-puncture (intervention group) and the other for the control group (no intervention).” (Page 14) | **Some concerns:**  Blinding cannot be performed.  There is ‘No information’ on whether any deviations arose because of the trial context. | **Some concerns:**  Blinding cannot be performed.  And ‘No information’ on whether the important non-protocol interventions were balanced across intervention groups. | **Low risk:**  No dropouts were reported**.** | **Low risk:**  " the study casts, was performed 1 month after the completion of the whole project by another researcher who was blind to the intervention group.” (Page 14)  The method of measuring the outcome is appropriate and the outcome assessor was blind. | **Low risk:**  No information about the registration protocol was mentioned. But the reported outcomes in the result section seemed to be corresponding with the pre-defined outcomes aforesaid in the method section. | **Some concerns** |
| **Raj 2020** | **Low risk:**  “Computer-generated randomization and quadrant allocation was carried out.” (Page e20) | **Some concerns:**  Blinding cannot be performed.  There is ‘No information’ on whether any deviations arose because of the trial context. | **Some concerns:**  Blinding cannot be performed.  And ‘No information’ on whether the important non-protocol interventions were balanced across intervention groups. | **Some concerns:**  The data availability from 95% of the participants would often be enough. In this study, the data availability was 80% (3 patients dropped out).  “Fig. 5 Consort flow diagram” (Page e23) | **High risk:**  “The rate of canine retraction was measured by estimating the change in the distance between the mesial aspect of the molar tube slot and the distal aspect of the canine bracket, measured intraorally by digital vernier calipers (Mitutoyo)” (Page e21)  This method has poor validity. Moreover, no details of the blinding of outcome assessors were reported. | **Low risk:**  No information about the registration protocol was mentioned. But the reported outcomes in the result section seemed to be corresponding with the pre-defined outcomes aforesaid in the method section. | **High risk** |
| **Sharma 2020** | **Some concerns:**  The method used for randomization was not reported.  "This study is a single centre, prospective, randomized clinical trial.” (Page 2) | **Some concerns:**  “Blinding of both patient and operator to the intervention was impossible.” (Page 4)  There is ‘No information’ on whether any deviations arose because of the trial context. (Page 5) | **Some concerns:**  “Blinding of both patient and operator to the intervention was impossible.” (Page 4)  And ‘No information’ on whether the important non-protocol interventions were balanced across intervention groups | **Low risk:**  “2 patients were dropped out and one patient was excluded. Therefore, a total number of 14 patients was evaluated.” (Page 7)  Based on the sample size calculation, 10 patients were required and the number was increased to 17 in order to compensate any possible dropout. | **Low risk:**  “the investigator who analysed the study models was blinded to the side at which the surgical intervention was performed. All data were labelled with numbers and sent to the statistician, who was also blinded.” (Page 4)  The method of measuring the outcome is appropriate and the outcome assessor was blind. | **Low risk:**  No information about the registration protocol was mentioned. But the reported outcomes in the result section seemed to be corresponding with the pre-defined outcomes aforesaid in the method section. | **Some concerns** |
| **Sirri 2020** | **Low risk:**  “Simple randomisation was conducted by using computer-generated random numbers with an allocation ratio of 1:1. The allocation sequence was concealed using sequentially numbered, opaque, sealed envelopes.” (Page 2) | **Low risk:**  “Patients were treated by the principal author (MRS) who was completely blinded. Periodontal indices were evaluated in both the groups before (three days before brackets bonding) and after the orthodontic treatment (seven days after brackets’ debonding) by the same principal author (MRS). Corticision and initial wire insertion were accomplished by one of the co-authors (MYH) who was also blinded to the study. Blinding of participants was not applicable.” (Page 2) | **Low risk:**  “Patients were treated by the principal author (MRS) who was completely blinded. Periodontal indices were evaluated in both the groups before (three days before brackets bonding) and after the orthodontic treatment (seven days after brackets’ debonding) by the same principal author (MRS). Corticision and initial wire insertion were accomplished by one of the co-authors (MYH) who was also blinded to the study. Blinding of participants was not applicable.” (Page 3) | **Low risk:**  “All patients attended their appointments, and there were no dropouts during the study.” (Page 3) | **Low risk:**  " blinding was applied for the outcomes’ assessor, who was one of the co-authors (ASB).” (Page 2)  The method of measuring the outcome is appropriate and the outcome assessor was blind. | **Some concerns:**  The protocol for the study was registered on clinical trial.gov with study ID: (NCT03310086), and the outcomes mentioned in the protocol have been reported (Except for the changes in lower teeth torque and tipping). Moreover, the periodontal assessment variable reported in the article was not mentioned in the protocol. | **Some concerns** |
| **Teh 2020** | **Low risk:**  “Thirty participants were randomly allocated into three equal groups using a simple random pattern generated online (Random.org). The sequence was then concealed in sealed individual envelopes with a unique identification number and revealed upon enrollment of  patients into the trial.” (Page 775, 776) | **Some concerns:**  Blinding cannot be performed.  There is ‘No information’ on whether any deviations arose because of the trial context. | **Some concerns:**  Blinding cannot be performed.  And ‘No information’ on whether the important non-protocol interventions were balanced across intervention groups. | **Low risk:**  “Thirty subjects were recruited, randomized and distributed into three groups of 10 subjects based on the MOP intervals of 4, 8, and 12 weeks. At the end of the trial, the CBCT data of only 24 participants were collected.” (Page 777)  Based on the sample size calculation, 24 patients were required and the number was increased to 30 in order to compensate any possible dropout. | **Low risk:**  “The CBCT files were labeled randomly by a research assistant for blinding of data during analysis by another examiner.” (Page 777)  The method of measuring the outcome is appropriate and the outcome assessor was blind. | **Low risk:**  The protocol for the study was registered on clinical trial.gov with study ID: (NCT03924726). | **Some concerns** |
| **Alqadasi 2021** | **Low risk:**  “The participants (in the MOPs or Piezo groups) and sides (experimental or control) were randomly allocated using a computer-generated random list simple method (https://www.random.org/). The program chose 24 random numbers divided into two groups. These numbers were put into opaque sealed envelopes determining the main category first and another small envelope inside determining whether the right or the left side will be used as experimental side.” (Page 6) | **Some concerns:**  “The blinding of both patients and clinicians was not possible during the treatment phase.” (Page 7)  There is ‘No information’ on whether any deviations arose because of the trial context. (Page 5) | **Some concerns:**  “The blinding of both patients and clinicians was not possible during the treatment phase.” (Page 7)  And ‘No information’ on whether the important non-protocol interventions were balanced across intervention groups | **Low risk:**  “three patients dropped out of the treatment, two patients in MOPs and one in Piezo group.” (Page 9)  “Nearly all” the outcome data is available. | **Low risk:**  “blinding was possible during the measurement stage and was accomplished using digital coding of the CBCT and the digital models. Decoding was performed before submitting the data for statistical analysis.” (Page 7)  The method of measuring the outcome is appropriate and the outcome assessor was blind. | **Low risk:**  No information about the registration protocol was mentioned. But the reported outcomes in the result section seemed to be corresponding with the pre-defined outcomes aforesaid in the method section. | **Some concerns** |
| **Bhaskaran 2021** | **Low risk:**  “This was a split-mouth design wherein 16 sites were selected and divided randomly into test (PAOO with PRF membrane) and control (PAOO without PRF membrane) sites by “lottery method.” (Page 2) | **Some concerns:**  Blinding cannot be performed.  There is ‘No information’ on whether any deviations arose because of the trial context. | **Some concerns:**  Blinding cannot be performed.  And ‘No information’ on whether the important non-protocol interventions were balanced across intervention groups. | **Low risk:**  No dropouts were reported.  “Fig. 3 CONSORT chart” (Page 5) | **Some concerns:**  No details of blinding of outcome assessors were reported.  The method of measuring the outcome is appropriate**.** | **Low risk:**  The protocol for the study was registered at WHO – Clinical Trials Registry – India with study ID: (CTRI/2018/11/016450). | **Some concerns** |
| **Çağlı Karci 2021** | **Low risk:**  “The randomization was performed via coin tosses to prevent selection bias... a randomly selected side of the maxillary arch received PRF injection in the PRF group and a piezocision technique in the piezocision group. The other side of the maxillary arch served as the control in both groups.” (Page 30) | **Some concerns:**  Blinding cannot be performed.  There is ‘No information’ on whether any deviations arose because of the trial context. | **Some concerns:**  Blinding cannot be performed.  And ‘No information’ on whether the important non-protocol interventions were balanced across intervention groups. | **Low risk:**  “There was no patient exclusion or loss during the study period.” (Page 32, 33) | **Some concerns:**  " All procedures and measurements were undertaken by the same researcher (I.C¸ .K.).” (Page 32)  The method of measuring the outcome is appropriate but the outcome assessor was not blind. | **Low risk:**  No information about the registration protocol was mentioned. But the reported outcomes in the result section seemed to be corresponding with the pre-defined outcomes aforesaid in the method section. | **Some concerns** |
| **Farag 2021** | **Low risk:**  “Assignment of patients and the choice of the side of intervention were done through a computer-generated randomization technique (www.random.org).” (Page 114) | **Some concerns:**  Blinding cannot be performed.  There is ‘No information’ on whether any deviations arose because of the trial context. | **Some concerns:**  Blinding cannot be performed.  And ‘No information’ on whether the important non-protocol interventions were balanced across intervention groups. | **Low risk:**  No dropouts were reported. | **High risk:**  “Data for the evaluation of each intervention were collected by direct intraoral measurements” (Page 115)  This method has poor validity. Moreover, no details of the blinding of outcome assessors were reported. | **Low risk:**  No information about the registration protocol was mentioned. But the reported outcomes in the result section seemed to be corresponding with the pre-defined outcomes aforesaid in the method section. | **High risk** |
| **Fernandes 2021** | **Low risk:**  "randomization by block (definition of the group and the decision of the experimental or control sides) was performed by Quick Calcs (Graph-Pad Software, Inc., La Jolla, CA) in a randomization center, by a person not associated to the patients’ recruitment center.” (Page 6) | **Some concerns:**  “Blinding of the surgeon (R.G.H.) and participants was not possible.” (Page 6)  There is ‘No information’ on whether any deviations arose because of the trial context. (Page 5) | **Some concerns:**  “Blinding of the surgeon (R.G.H.) and participants was not possible.” (Page 6)  And ‘No information’ on whether the important non-protocol interventions were balanced across intervention groups | **Low risk:**  "There were 4 patient dropouts.” (Page 7)  The resulting dropouts remained within the compensation limits that were taken into account during the sample size calculation. | **Low risk:**  “For the digital models’ measurements, the operator (D.F.F.) was not aware of the patients group, as well as whether the side was experimental or control.” (Page 6)  The method of measuring the outcome is appropriate and the outcome assessor was blind. | **Some concerns:**  The protocol for the study was registered on clinical trial.gov with study ID: (NCT03089996) ), and the outcomes mentioned in the protocol have been reported (patient Pain and discomfort and first molars anchorage loss). | **Some concerns** |
| **Jaiswal 2021** | **Low risk:**  “Random numbers were generated in the permuted random block size of 2 using the Research Randomizer software (Research Randomizer, Version 4, Urbaniak, G.C., & Plous, S.) by the investigator AJ. The numbers were concealed in opaque envelopes and kept in a box. Each patient was then asked to pick a sealed envelope to assign the second MOP to either the right or left side, executed separately without any role of primary clinical investigators, shuffled every time before picking.” (Page 417) | **Some concerns:**  Blinding cannot be performed.  There is ‘No information’ on whether any deviations arose because of the trial context. | **Some concerns:**  Blinding cannot be performed.  And ‘No information’ on whether the important non-protocol interventions were balanced across intervention groups. | **Low risk:**  “One patient was excluded after intervention owing to miniscrew implant failure.” (Page 418)  “Nearly all” the outcome data is available. | **Low risk:**  “However, the extracted data was coded during collection and analysis to ensure blinding.” (Page 417)  The method of measuring the outcome is appropriate and the outcome assessor was blind. | **Low risk:**  The protocol for the study was registered in the Clinical trial registry of India (CTRI/2018/05/013953 and CTRI/2018/05/014121). | **Some concerns** |
| **Ozkan 2021** | **Low risk:**  "a random allocation software (RAND function; Microsoft Excel  2010, Redmond, WA, USA) was used to allocate the patients into either the control (0) or the experimental group (1). Each patient was then asked to choose an opaque, brown envelope.” (Page 160) | **Some concerns:**  “The clinical procedures were managed by a single operator (T.O.). All the patients were blinded to the procedure as they had to fill the VAS for both the left and right sides of the mouth.” (Page 160)  There is ‘No information’ on whether any deviations arose because of the trial context. (Page 5) | **Some concerns:**  The clinical procedures were managed by a single operator (T.O.). All the patients were blinded to the procedure as they had to fill the VAS for both the left and right sides of the mouth.” (Page 160)  And ‘No information’ on whether the important non-protocol interventions were balanced across intervention groups | **Low risk:**  "A total of 24 patients were included in this study, and all of them completed the treatment.” (Page 161) | **Low risk:**  "the examiner assessing the study casts was blinded to the participant's assigned intervention” (Page 160)  The method of measuring the outcome is appropriate and the outcome assessor was blind. | **Low risk:**  No information about the registration protocol was mentioned. But the reported outcomes in the result section seemed to be corresponding with the pre-defined outcomes aforesaid in the method section. | **Some concerns** |
| **Qi 2021** | **Low risk:**  "Patients were randomly assigned to the control group (collagen barrier membrane) or test group (CGF). Sealed envelopes were used to perform randomization with an equal number of envelopes for every group.” (Page 3) | **Some concerns:**  Blinding cannot be performed.  There is ‘No information’ on whether any deviations arose because of the trial context. | **Some concerns:**  Blinding cannot be performed.  And ‘No information’ on whether the important non-protocol interventions were balanced across intervention groups. | **Some concerns:**  " A total of 40 patients completed the study after 4 dropouts” (Page 5)  Based on sample size calculation "a minimum of 22 patients was needed for each group so as to obtain 80% power in our study after considering 10% dropouts." (Page 2) | **Low risk:**  "Group allocation was revealed just before surgery and remained blinded for the evaluating investigator for data collection, analysis, and processing during the project’s analytical stage” (Page 3)  The method of measuring the outcome is appropriate and the outcome assessor was blind. | **Low risk:**  The protocol for the study was registered in Chinese Clinical Trial Registry (http:// www. chictr. org. cn/ index. aspx) under number (ChiCTRINR17013346). | **Some concerns** |
| **Raghav 2021** | **Low risk:**  "a random allocation was done by block randomization method with a block size of 6 in 1:1 ratio of allocation. The concealment of treatment modality which would be performed was done in sequentially numbered, sealed, opaque envelopes which were shuffled by an independent investigator, and chosen by the patient.” (Page 186) | **Some concerns:**  “Blinding was not possible at the time of intervention for the subjects and clinicians.” (Page 186)  There is ‘No information’ on whether any deviations arose because of the trial context. (Page 5) | **Some concerns:**  “Blinding was not possible at the time of intervention for the subjects and clinicians.” (Page 186)  And ‘No information’ on whether the important non-protocol interventions were balanced across intervention groups | **Low risk:**  "2 subjects from the experimental and 3 from control group were lost in follow up.” (Page 188)  The resulting dropouts remained within the compensation limits that were taken into account during the sample size calculation. | **Low risk:**  "Blinding was done at the analytical level where all the measurements and analysis on the coded models were done by another investigator, who was blinded for the both groups” (Page 186)  The method of measuring the outcome is appropriate and the outcome assessor was blind. | **Low risk:**  The protocol for the study was registered at www.ctri.nic.in with CTRI with study ID: (CTRI/2019/03/018140) | **Some concerns** |
| **Shahrin 2021** | **Low risk:**  "A random number table was employed. Cards were written with numbers and set in opaque sealed envelopes,... Sequentially numbered opaque, sealed envelopes were used for concealed randomization of group allocation to minimize the risk of bias.” (Page 4) | **Some concerns:**  “It is not feasible to blind the participants and the operator during the intervention.” (Page 4)  There is ‘No information’ on whether any deviations arose because of the trial context. (Page 5) | **Some concerns:**  “It is not feasible to blind the participants and the operator during the intervention.” (Page 4)  And ‘No information’ on whether the important non-protocol interventions were balanced across intervention groups | **Low risk:**  "1 participant dropped out from the MOPs group because of pregnancy, and 1 was lost in the control group because the patient failed to attend follow-up records for 2 consecutive months.” (Page 5)  “Nearly all” the outcome data is available. | **Low risk:**  "the examiner assessing the study casts was blinded to the participant's assigned intervention” (Page 4)  The method of measuring the outcome is appropriate and the outcome assessor was blind. | **Some concerns:**  The protocol for the study was registered ISRCTN registry with study ID: (ISRCTN15080404), and the outcomes mentioned in the protocol have been reported (Except for the degree of root resorption and pain perception) | **Some concerns** |
| **Sirri 2021** | **Low risk:**  "Simple randomization was performed by one of the academic staff (not involved in the current study) at the Department of Orthodontics using a computer-generated random number sequence, with an allocation ratio of 1: 1. The allocation sequence was hidden using closed, opaque, numbered envelopes.” (Page 4) | **Some concerns:**  “The blindness of participants was not applicable in this trial. Therefore, the blindness was applied only during data analysis.” (Page 4)  There is ‘No information’ on whether any deviations arose because of the trial context. (Page 5) | **Some concerns:**  “The blindness of participants was not applicable in this trial. Therefore, the blindness was applied only during data analysis.” (Page 4)  And ‘No information’ on whether the important non-protocol interventions were balanced across intervention groups | **Low risk:**  No dropouts were reported.  “Figure 1: CONSORT flow diagram of patients' recruitment, follow-up, and entry into data analysis” (Page 3) | **Low risk:**  "the blindness was applied only during data analysis.” (Page 4)  The method of measuring the outcome is appropriate and the outcome assessor was blind. | **Low risk:**  The protocol for the study was registered on clinical trial.gov with study ID: (NCT04601662). | **Some concerns** |
| **Al-Ibrahim 2022** | **Low risk:**  "SPSS for Windows, version 20 (IBM Corporation, Armonk, NY, USA) was used to determine the allocation of patients among the 3 groups, using a set of random numbers with an allocation ratio of 1:1:1.” (Page 4) | **Some concerns:**  “It was not possible to make the treatment procedures blind for either patients or practitioners.” (Page 5)  There is ‘No information’ on whether any deviations arose because of the trial context. (Page 5) | **Some concerns:**  “It was not possible to make the treatment procedures blind for either patients or practitioners.” (Page 5)  And ‘No information’ on whether the important non-protocol interventions were balanced across intervention groups | **Low risk:**  "No patient was lost to follow up; therefore, 57 patients were included in the data analysis.” (Page 7) | **Low risk:**  "so blinding was limited to data analysis” (Page 5)  The method of measuring the outcome is appropriate and the outcome assessor was blind. | **Low risk:**  The protocol for the study was registered on clinical trial.gov with study ID: (NCT04950829). | **Some concerns** |
| **Bavikati 2022** | **Some concerns:**  The method used for randomization was not reported.  "The study was a split mouth design; the experimental side was allocated by randomization.” (Page 256) | **Some concerns:**  Blinding cannot be performed.  There is ‘No information’ on whether any deviations arose because of the trial context. | **Some concerns:**  Blinding cannot be performed.  And ‘No information’ on whether the important non-protocol interventions were balanced across intervention groups. | **Low risk:**  No dropouts were reported. | **Low risk:**  "All cast measurements were made using a digital Vernier calliperby one operator who was blinded about the study” (Page 257)  The method of measuring the outcome is appropriate and the outcome assessor was blind. | **Low risk:**  No information about the registration protocol was mentioned. But the reported outcomes in the result section seemed to be corresponding with the pre-defined outcomes aforesaid in the method section. | **Some concerns** |
| **Bolat Gümüş 2022** | **Low risk:**  "Randomization for either the right or left side was achieved the flip of the coin.” (Page 3) | **Some concerns:**  “Randomization was not blinded, as patients and residents were aware of the situation.” (Page 3)  There is ‘No information’ on whether any deviations arose because of the trial context. | **Some concerns:**  “Randomization was not blinded, as patients and residents were aware of the situation.” (Page 3)  And ‘No information’ on whether the important non-protocol interventions were balanced across intervention groups. | **Low risk:**  No dropouts were reported. | **Some concerns:**  "All measurements were performed by the same resident (E.K.)” (Page 4)  The method of measuring the outcome is appropriate but the outcome assessor was not blind. | **Low risk:**  No information about the registration protocol was mentioned. But the reported outcomes in the result section seemed to be corresponding with the pre-defined outcomes aforesaid in the method section. | **Some concerns** |
| **Hasaneen 2022** | **Some concerns:**  The method used for randomization was not reported.  "Patients were randomly divided into two equal groups.” (Page 153) | **Some concerns:**  Blinding cannot be performed.  There is ‘No information’ on whether any deviations arose because of the trial context. | **Some concerns:**  Blinding cannot be performed.  And ‘No information’ on whether the important non-protocol interventions were balanced across intervention groups. | **Low risk:**  No dropouts were reported. | **Some concerns:**  No details of blinding of outcome assessors were reported.  The method of measuring the outcome is appropriate**.** | **Low risk:**  No information about the registration protocol was mentioned. But the reported outcomes in the result section seemed to be corresponding with the pre-defined outcomes aforesaid in the method section. | **High risk** |
| **Hawkins 2022** | **Low risk:**  " Using www.randomisation.com, a restricted 1:1 randomization list was generated. Allocation concealment was achieved with opaque, sealed envelopes created and kept by a person not involved in any clinical contact with the patients.” (Page 10) | **Some concerns:**  “Blinding of patients and operators was not possible.” (Page 10)  There is ‘No information’ on whether any deviations arose because of the trial context. | **Some concerns:**  “Blinding of patients and operators was not possible.” (Page 10)  And ‘No information’ on whether the important non-protocol interventions were balanced across intervention groups. | **Low risk:**  "Two patients were excluded from the study.” (Page 11)  “Nearly all” the outcome data is available. | **Low risk:**  "The examiner performing the dental cast measurements was blinded” (Page 10)  The method of measuring the outcome is appropriate and the outcome assessor was blind. | **Low risk:**  No information about the registration protocol was mentioned. But the reported outcomes in the result section seemed to be corresponding with the pre-defined outcomes aforesaid in the method section. | **Some concerns** |
| **Jaber 2022** | **Low risk:**  "A computer-generated list of random numbers was exported by Minitab (version 17, Minitab, LLC, State College, Pa, USA) assigning each side of the upper jaw (left or right) to the experimental group in an allocation ratio of 1:1.” (Page 3) | **Some concerns:**  “Blinding of personnel and participants were not applicable.” (Page 3)  There is ‘No information’ on whether any deviations arose because of the trial context. | **Some concerns:**  “Blinding of personnel and participants were not applicable.” (Page 3)  And ‘No information’ on whether the important non-protocol interventions were balanced across intervention groups. | **Low risk:**  "No patient was lost to follow-up.” (Page 5) | **Low risk:**  "blinding was applied only for the outcomes’ assessor” (Page 3)  The method of measuring the outcome is appropriate and the outcome assessor was blind. | **Low risk:**  The protocol for the study was registered on clinical trial.gov with study ID: (NCT04316403). | **Some concerns** |
| **Khlef 2022** | **Low risk:**  "Simple randomization was carried out by an academic colleague using random numbers generated by the computer with a 1 :1 allocation ratio. -e allocation sequence was hidden using sequentially numbered, opaque, sealed envelopes.” (Page 4) | **Some concerns:**  “Blinding was impossible for both patients and investigators carrying out the clinical procedures.” (Page 5)  There is ‘No information’ on whether any deviations arose because of the trial context. | **Some concerns:**  “Blinding was impossible for both patients and investigators carrying out the clinical procedures.” (Page 5)  And ‘No information’ on whether the important non-protocol interventions were balanced across intervention groups. | **Low risk:**  "One participant had missed the follow-up appointments in each group, leaving 18 patients per group for the data analysis stage.” (Page 6)  “Nearly all” the outcome data is available. | **Low risk:**  "blinding was performed only for the assessor of outcomes” (Page 5)  The method of measuring the outcome is appropriate and the outcome assessor was blind. | **Low risk:**  The protocol for the study was registered on clinical trial.gov with study ID: (NCT04847492). | **Some concerns** |
| **Li 2022** | **Low risk:**  "A 1:1 split-mouth design was used in which MOPs were applied to one side of the mouth for each subject and the other side was used as control. The experimental side was chosen using a randomization table by an investigator not involved in patient management. Allocation concealment was performed by a staff member not directly involved with the trial. Separate sealed and opaque envelopes.” (Page 4) | **Some concerns:**  “Blinding was not possible during the clinical procedure.” (Page 4)  There is ‘No information’ on whether any deviations arose because of the trial context. | **Some concerns:**  “Blinding was not possible during the clinical procedure.” (Page 4)  And ‘No information’ on whether the important non-protocol interventions were balanced across intervention groups. | **Low risk:**  "no patient was lost to follow-up.” (Page 4) | **Low risk:**  "prior to study model measurements, the author doing the measurements on study models (JL) was blinded” (Page 4)  The method of measuring the outcome is appropriate and the outcome assessor was blind. | **Low risk:**  "Trial was not registered” (Page 1)  However, the reported outcomes in the result section seemed to be corresponding with the pre-defined outcomes aforesaid in the method section. | **Some concerns** |
| **Saad 2022** | **Some concerns:**  The method used for randomization was not reported.  "Twenty two patients were randomly divided into two groups.” (Page 147) | **Some concerns:**  Blinding cannot be performed.  There is ‘No information’ on whether any deviations arose because of the trial context. | **Some concerns:**  Blinding cannot be performed.  And ‘No information’ on whether the important non-protocol interventions were balanced across intervention groups. | **Low risk:**  "Clinically; 22 patients had successful healing and complete canine retraction.” (Page 149) | **Some concerns:**  No details of blinding of outcome assessors were reported.  The method of measuring the outcome is appropriate**.** | **Low risk:**  No information about the registration protocol was mentioned. But the reported outcomes in the result section seemed to be corresponding with the pre-defined outcomes aforesaid in the method section. | **High risk** |
| **Simre 2022** | **Low risk:**  "A split-mouth design was employed and corticotomy was performed using piezo (study group) on one random side and bur (control group) on the contralateral side in maxilla as well as mandible using a computer-generated randomization list.” (Page 183) | **Some concerns:**  Blinding cannot be performed.  There is ‘No information’ on whether any deviations arose because of the trial context. | **Some concerns:**  Blinding cannot be performed.  And ‘No information’ on whether the important non-protocol interventions were balanced across intervention groups. | **Low risk:**  "All 24 subjects completed the study.” (Page 183) | **Low risk:**  " All the measurements were recorded pre- and post-operatively by an Orthodontist who was blinded to the study” (Page 183)  The method of measuring the outcome is appropriate and the outcome assessor was blind. | **Low risk:**  No information about the registration protocol was mentioned. But the reported outcomes in the result section seemed to be corresponding with the pre-defined outcomes aforesaid in the method section. | **Some concerns** |
|  |  |  |  |  |  |  |  |
