# Supplementary material for: The transparency of reporting 'harms' encountered with the surgically assisted acceleration of orthodontic tooth movement in the published randomized controlled trials: a meta-epidemiological study
Source: Prog Orthod. 2023 Mar 21;24:11. doi: 10.1186/s40510-023-00457-4 (PMC10027979; doi:10.1186/s40510-023-00457-4)
Supplement: Supplementary file 6 — Additional file 6: Table S4. Responses from authors regarding the SAAO-related harms. [file 40510_2023_457_MOESM6_ESM.docx]

| **Supplementary Table 4:** Responses from authors regarding the SAAO-related harms | | | | | | | | | |
| --- | --- | --- | --- | --- | --- | --- | --- | --- | --- |
| **Study** | **setting** | **Publication journal** | | **Methods** | | **Participants** | **Orthodontic procedure** | **Invasiveness of the SAAO** | **Reported harm** related to the SAAO |
|  |  | Title | Quartile | Study  design | Treatment  comparison | Patients (M/F)  Age (years) |  |  |  |
| **Shoreibah et al. 2012** | Egypt | AL Azhar Dental Journal for girls | Not indexed | RCT, 2-Arms PG | Corticotomy vs. NAC | Patients (M/F): 20 (3\ 17)  Age (years): 18.4- 25 | Decrowding of lower anterior  teeth | Invasive | No harm reported. |
| **Al-Naoum et al. 2014** | Syria | Journal of Oral and Maxillofacial Surgery | Q2* | RCT, SMD | Corticotomy vs. NAC | Patients (M/F): 30 (15\ 15)  Age (years): 15- 24 | Maxillary canine retraction | Invasive | - Severe gingival inflammation at the edges of the surgical incision in one case.  - Numbness in the corner of the upper lip in one case. |
| **Al-Naoum et al. 2015** | Syria | International Arab Journal of Dentistry | Not indexed | RCT, 2-Arms PG | PAOO vs. NAC | Patients (M/F): 30 (13\ 17)  Age (years): 16- 24 | Decrowding of the anterior teeth | Invasive | No harm related to periodontium or dental pulp was observed. |
| **Aristizabal et al. 2016** | Colombia | International journal of odontostomatology | Not indexed | RCT, 2-Arms PG | PAOO vs. NAC | Patients (M/F): 10 (10\ 0)  Age (years): 18- 40 | Decrowding of the anterior teeth | Invasive | No harm reported. |
| **Abdelhameed and Refai, 2018** | Egypt | Open Access Macedonian Journal of Medical Sciences | Q3* | RCT, COMP | (MOPs /NAC) vs. (LLLT /NAC) vs. (MOPs+ LLLT /NAC) | Patients (M/F): 30 (NR\ NR)  Age (years): 18- 40 | Maxillary canine retraction | Minimally invasive | -Slight redness.  -Mild pain.  -Edema. |
| **Aboalnaga et al. 2019** | Egypt | Progress in Orthodontics | Q1* | RCT, SMD | MOPs vs. NAC | Patients (M/F): 18 (0\ 18)  Age (years): 15- 25 | Maxillary canine retraction | Minimally invasive | -Mild swelling in 3 patients.  -Mild bruising in the area of the MOPs in a few patients.  -Mucosal perforation tear especially in the most apical MOP in 5 patients. |
| **Agrawal et al. 2019** | India | Saudi Dental Journal | Q1* | RCT, SMD | Corticotomy vs. MOPs | Patients (M/F): 10 (NR\ NR)  Age (years): 18-25 | Maxillary canine retraction | Combination of IP and MIP in PG or SMDs | No harm related to SAAO was observed. |
| **Kumar et al. 2019** | India | Journal of Orthodontic Science | Q2* | RCT, 2-Arms PG | Corticotomy vs. NAC | Patients (M/F): 20 (NR\ NR)  Age (years): 15-30 | Maxillary and Mandibular canine retraction | Invasive | No harm reported. |
| **Singh et al. 2019** | India | International Journal Of Periodontics And Restorative Dentistry | Q2* | RCT, 2-Arms PG | PAOO vs. NAC | Patients (M/F): 30 (NR\ NR)  Age (years): 18- 40 | EN-masse retraction | Invasive | No harm reported. |
| **Sivarajan et al. 2019** | Malaysia | Angle Orthodontist | Q1* | RCT, COMP | (MOP 4-WMax/8-WMan/NAC) vs. (MOP 8-WMax /12- WMan/NAC) vs. (MOP 12-WMax /4- WMan /NAC) | Patients (M/F): 30 (7\ 23)  Age (years): ≥18 | Maxillary and Mandibular canine retraction | Minimally invasive | No harm reported. |
| **Abdarazik et al. 2020** | Egypt | AL Azhar Dental Journal for girls | Not indexed | RCT, COMP | (FTMPF\ NAC) vs. (LLLT\ NAC) | Patients (M/F): 32 (0\ 32)  Age (years): 15-20 | Maxillary canine retraction | Invasive | -Discomfort in most patients.  - Edema lasted for 2 days in a few patients.  -Tooth sensitivity in one patient.  - No radiographic bone loss was recorded in any of the patients. |
| **Alfawal et al. 2020** | Syria | Dental and Medical Problems | Q3* | RCT, COMP | (Piezocision \ NAC) vs. (LAFC \ NAC) | Patients (M/F): 32 (13\ 19)  Age (years): 16-29 | Maxillary canine retraction | Invasive Minimally invasive | -In piezocision group:  One patient suffered from severe inflammation and hyperemia with oozing of pus in one incision site, and one patient complained of the unsightly scarring appearance of the cuts.  -In LAFC group:  View of scarring around the canine in one case. |
| **Asif et al. 2020** | Malaysia | American Journal of Orthodontics and Dentofacial Orthopedics | Q1* | RCT, COMP | (MOP 4W /NAC) vs. (MOP 8W/NAC) vs. (MOP 12W/NAC) | Patients (M/F): 30 (NR\ NR)  Age (years): ≥18 | Mandibular canine retraction | Minimally invasive | No harm reported. |
| **El Mahlawy et al. 2020** | Egypt | AL Azhar Dental Journal for girls | Not indexed | RCT, 2-Arms PG | Piezocision without BG vs. Piezocision with BG | Patients (M/F): 20 (0\ 20)  Age (years): 18- 25 | EN-masse retraction | Minimally invasive | Postoperative swelling and pain in some patients for a few days. |
| **Fattori et al. 2020** | Brazil | Angle Orthodontist | Q1* | RCT, 2-Arms PG | MOPs vs. NAC | Patients (M/F): 18 (7\ 11)  Age (years): 18 - 36 | EN-masse retraction | Minimally invasive | - Bleeding (but just the expected).  -Post-surgery pain.  - Some patients were afraid and avoided the perforations (as MOPs were repeated every month). |
| **Omidkhoda et al. 2020** | Iran | Turkish Journal of Orthodontics | Q3* | RCT, SMD | Piezo-puncture vs. NAC | Patients (M/F): 17 (7\ 10)  Age (years): 14-30 | Maxillary canine retraction | Minimally invasive | No harm reported. |
| **Raj et al. 2020** | India | International Journal Of Periodontics And Restorative Dentistry | Q2* | RCT, SMD | Piezocision vs. NAC | Patients (M/F): 20 (6\ 14)  Age (years): 20-25 | Maxillary canine retraction | Minimally invasive | No harm reported. |
| **Sirri et al. 2020** | Syria | Journal of Clinical and Diagnostic Research | Q3* | RCT, 2-Arms PG | Corticision vs. NAC | Patients (M/F): 60 (19\ 41)  Age (years): 21.40±1.63 | Decrowding of the Lower anterior teeth | Minimally invasive | Post-surgery dizziness and hypotension in one case. |
| **Teh et al. 2020** | Malaysia | Angle Orthodontist | Q1* | RCT, COMP | (MOP 4W /NAC) vs. (MOP 8W/NAC) vs. (MOP 12W/NAC) | Patients (M/F): 30 (NR\ NR)  Age (years): 19-27 | Mandibular canine retraction | Minimally invasive | -Bleeding (but just the expected).  -Discomfort in very few patients. |
| **Sirri et al. 2021** | Syria | International Orthodontics | Q3* | RCT, 2-Arms PG | Corticision vs. NAC | Patients (M/F): 52 (14\ 38)  Age (years): 18-24 | Decrowding of the Lower anterior teeth | Minimally invasive | No harm reported. |
| **Bavikati et al. 2022** | India | Medicine & Pharmacy Reports | Q2* | RCT, 2-Arms PG | MOPs vs. NAC | Patients (M/F): 22 (0\ 22)  Age (years): 18.4- 25 | Maxillary canine retraction | Minimally invasive | No harm reported. |
| **Simre et al. 2022** | India | Journal of Oral Biology and Craniofacial Research | Q2* | RCT, SMD | Flapless corticotomy using piazo vs. Flapless corticotomy using bur | Patients (M/F): 24 (13\ 11)  Age (years): 14- 25 | Maxillary and Mandibular canine retraction | Minimally invasive | - One patient developed a significant hematoma in the lower lip while applying corticotomy using bur. |
| **SAAO:** surgically-assisted accelerated orthodontics; **RCT:** Randomized clinical trial; **SMD:** Split-mouth design; **PG:** Parallel group; **COMP:** Compound design (it consists of both parallel and split-mouth); **NAC:** Non-Accelerated Control; **MOPs:** Micro-osteoperforations; **WMax**: weekly maxilla; **WMan**: weekly mandible; **W**: weeks; **BG:** Bone graft; **PAOO:** Periodontally accelerated osteogenic orthodontics; **FTMPF:** full-thickness mucoperiosteal flap; **LLLT:** low-level laser therapy; **LAFC**: Laser-assisted flapless corticotomy; **NR:** Not reported; **IP**: Invasive procedure; **MIP**: Minimally invasive procedure  * Indexed according to Scopus® database, ** not indexed by both Scopus® and Web of Science™ databases | | | | | | | | | |
